# Facile Design of Highly Stretchable and Conductive Crumpled Graphene/NiS_2_ Films for Multifunctional Applications

**DOI:** 10.1002/smtd.202401965

**Published:** 2025-01-09

**Authors:** Kangwei Weng, Qiji Jing, Jindong Gao, Weiguo Wang, Chen Zhang, Jun Wang, Huanyu Cheng, Cheng Zhang

**Affiliations:** ^1^ Fujian Provincial Key Laboratory of Functional Marine Sensing Materials College of Material and Chemical Engineering Minjiang University Fuzhou 350108 P. R. China; ^2^ School of Materials Science and Engineering Fujian University of Technology Fuzhou Fujian 350506 P. R. China; ^3^ Department of Engineering Science and Mechanics Materials Research Institute Pennsylvania State University University Park Pennsylvania 16802 USA

**Keywords:** crumpled graphene/NiS_2_ nanocomposite, micro‐supercapacitor arrays, triboelectric nanogenerator, wearable biophysical sensors

## Abstract

The cost‐effective and scalable synthesis and patterning of soft nanomaterial composites with improved electrical conductivity and mechanical stretchability remains challenging in wearable devices. This work reports a scalable, low‐cost fabrication approach to directly create and pattern crumpled porous graphene/NiS_2_ nanocomposites with high mechanical stretchability and electrical conductivity through laser irradiation combined with electrodeposition and a pre‐strain strategy. With modulated mechanical stretchability and electrical conductivity, the crumpled graphene/NiS_2_ nanocomposite can be readily patterned into target geometries for application in a standalone stretchable sensing platform. By leveraging the electrical energy harvested from the kinetic motion from wearable triboelectric nanogenerator (TENG) and stored in micro‐supercapacitor arrays (MSCAs) to drive biophysical sensors, the system is demonstrated to monitor human motions, body temperature, and toxic gas in the exposed environment. The material selections, design strategies, and fabrication approaches from this study provide functional nanomaterial composites with tunable properties for future high‐performance bio‐integrated electronics.

## Graphical abstract

A scalable, low‐cost fabrication approach is reported to directly create and pattern crumpled porous graphene/NiS_2_ nanocomposites with high mechanical stretchability and electrical conductivity through laser irradiation combined with electrodeposition and a pre‐strain strategy. This novel synthetic approach holds promise for a variety of bio‐integrated electronic applications.

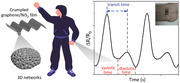

## 1 Introduction

With application opportunities in wearable/biomedical devices, bio‐integrated electrodes, human‐machine interfaces, and stretchable electronics have spurred rapid developments of mechanically soft materials and advanced fabrication technologies.^[^ ^1^ ^]^ A wide range of nanomaterials including metals,^[^ ^2^ ^]^ carbons,^[^ ^3^ ^]^ polymers,^[^ ^4^ ^]^ and metal oxides^[^ ^5^ ^]^ has been exploited by with varying fabrication techniques (e.g., bulk micromachining, 3D printing, molding, photonic sintering).^[^ ^6^ ^]^ In particular, porous laser‐induced graphene (LIG) foams with 3D networks prepared by irradiating a carbon source (e.g., polyimide, wood, cotton, cork) with a low‐cost laser exhibit high surface area, mechanical stiffness, chemical stability, and very high electrical and thermal conductivity.^[^ ^7^ ^]^ As a result, the LIG‐based stretchable devices such as electronic circuits, energy conversion and storage devices, sensors, and soft actuators demonstrate a stable output performance upon various mechanical deformations during a long‐term operation.^[^ ^8^ ^]^ In addition, the thermal vibration of the graphene lattice, edge instability and/or defect, and trapped solvent evaporation/removal of 3D porous LIG foams provide them with a high degree of structural and chemical tenability through surface coating or chemical functionalization.^[^ ^9^ ^]^ The hybrids of LIG foams with other nanomaterials such as metal/alloys or metal oxides/sulfides show short ions/electrons diffusion path, high strength and elasticity properties, and tunable structures/electrical properties while still maintaining easily accessible 3D network structure.^[^ ^3^ ^]^ Among metal compounds, nickel sulfides (e.g., NiS, NiS_2_, Ni_3_S_2_, Ni_7_S_6_, and Ni_9_S_8_) are low‐cost, earth‐abundant, environmental benign, and exhibit tunable properties.^[^ ^10^ ^]^ In particular, NiS_2_ characteristic electronic structure, high conductivity, large active site, and electrochemical activity are considered as an attractive candidate for wearable/biomedical devices.^[^ ^11^ ^]^ Therefore, hybrid LIG/NiS_2_ nanocomposites could take advantage of the high electronic conductivity from both the LIG foams and NiS_2_, the high electrochemical activity of NiS_2_, and the short ion diffusion pathway in the 3D porous LIG, resulting in enhanced charge (both electrons and ions) transfer process and electrochemical performance. Although several strategies such as spray coating and laser irradiation have been demonstrated to prepare the LIG‐based nanocomposites,^[^ ^12^, ^13^, ^14^ ^]^ it is still challenging to prepare the LIG‐based nanomaterials with desired morphology, crystal structure, composition, and physicochemical properties in a scalable approach. Furthermore, it is of great interest to develop an efficient and scalable approach to prepare LIG‐based nanomaterials that can load a higher mass of active materials and exhibit robust interfaces even upon mechanical deformations.

Crumpled structures of LIG and LIG‐based nanomaterial composites can be created as stretchable functional materials through the pre‐strain and release of the substrate or surface anchorage and/or high solvent surface tension during the transfer process.^[^ ^15^, ^16^ ^]^ Besides enhanced stretchability, these corrugated functional materials also show modulated conductivity and surface/interface structures (e.g., surface roughness, work functions, specific active sites, and wettability, among others). Because of the unfolding of the crumpled structure upon stretching, the well‐connected 3D graphene network still maintains intrinsic structure and conductivity for stable device performance. Crumpled LIG structures can be also combined with other stretchable structural designs (e.g., serpentine bridge‐island layout, helix shapes, kirigami/origami designs) to further enhance stretchability.^[^ ^17^ ^]^ Despite these advantages, it still remains elusive to balance of the conductivity and mechanical stretchability especially under extremely large mechanical strains over time, which mostly results from the poor interface between the LIG scaffold and loading functional nanomaterials.

This research demonstrates a low‐cost, scalable fabrication method to prepare crumpled graphene/NiS_2_ nanocomposite patterns with a highly stable interface through combined laser scribing, electrodeposition, and the pre‐strain strategy. With relatively uniform porous NiS_2_ nanoparticles directly grown on the 3D LIG conductive scaffold, the resulting crumpled graphene/NiS_2_ nanocomposite features good mechanical adhesion and electrical contact for improved electrical conductivity and mechanical stretchability. The facile fabrication and patterning approach allows the creation of a standalone stretchable device platform, consisting of a triboelectric nanogenerator (TENG), micro‐supercapacitor arrays (MSCAs), and varying biophysical sensors, entirely based on crumpled graphene/NiS_2_ nanocomposite. The human kinetic motion energy can be converted into electrical energy by the TENG and then stored into the MSCAs to drive highly sensitive biophysical sensors for monitoring (large or ultrasmall) human motions, body temperature, and toxic NO_2_ gas in the exposed environment. The cost‐efficient and scalable approach for fabricating crumpled porous patterns provides functional nanomaterials with adjustable surface structures and material properties for a new class of flexible/stretchable electronics.

## 2 Results and Discussion

### 2.1 Fabrication of the Patterning Crumpled Porous Graphene/NiS_2_ Nanocomposite Films

**Figure 1:**
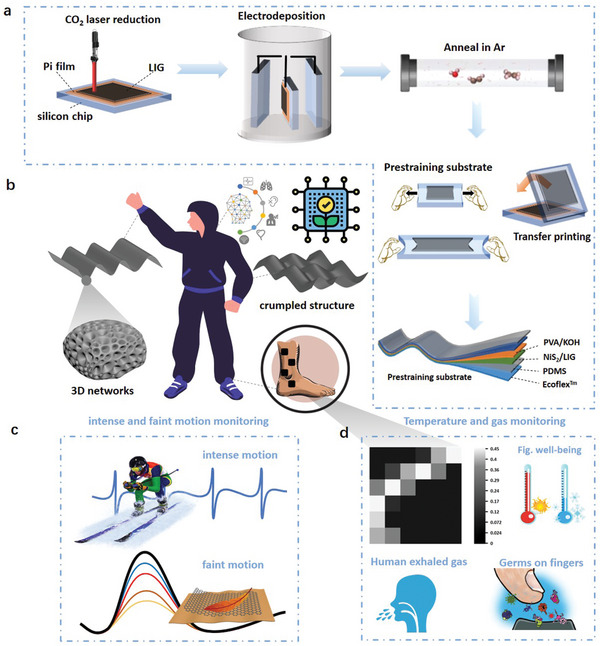
Design, fabrication, and application of stretchable and highly conductive crumpled porous graphene/NiS_2_ nanocomposite patterns. a) Schematic showing the fabrication process of the porous graphene/NiS_2_ nanocomposite through direct laser writing and the electrodeposition followed by transfer printing onto the pre‐strained elastomeric substrate. b) Seamless and conformal contact of the stretchable crumpled porous graphene/NiS2 nanocomposite with the human skin allows the application in c) human motion monitoring and d) biophysical sensing.

The crumpled graphene/NiS_2_ nanocomposite patterns with 3D networks are created through facile direct laser writing and electrodeposition, followed by transfer printing onto a pre‐strained elastomeric substrate. In brief, the interconnected porous laser‐induced graphene (LIG) patterns prepared by irradiation of a thin polyimide (PI) film with a CO_2_ laser (wavelength of 10.6 µm and laser power of 3.8 W) (**Figure** **1**a) can act as the working electrode for electrodeposition of the crystallized NiS_2_ nanoparticles directly on LIG without any polymeric binder and conductive additive. Following transfer printing and the pre‐strain strategy^[^ ^14^ ^]^ can then obtain crumpled porous LIG/NiS_2_ nanocomposite films, with well well‐controlled degree of crumple by the level of pre‐strain (Figure 1b). The highly crumpled nanocomposite exhibits high stretchability and mechanical robustness, along with improved electrical conductivity owing to increased interfacial interactions between nanocomposites and charge carriers. Compared to the previous synthesis strategies,^[^ ^12^, ^13^, ^14^ ^]^ this reported method also allows uniform coating of active materials on the surface and throughout the depth of the porous LIG skeleton (Figure S1, Supporting Information) to load a higher mass of active materials and ensure enhanced mechanical adhesion and electrical contact even upon mechanical deformations (Figure S2, Supporting Information). Additionally, this method allows facile control of the morphology, crystal structure, and physicochemical properties of the resulting LIG nanocomposites by simply adjusting the electrodeposition conditions such as the ions in the solution, deposition rates, electrodeposition time, temperatures, pH values, and additives during the electrodeposition. For instance, adjusting the atomic percentage of Ni and S in the solution can yield Ni_3_S_2_, NiS, NiS_2_, and Ni_x_S_6_ (Figure S3, Supporting Information), with the particle size and distribution density controlled by the electrodeposition time (Figure S4, Supporting Information). Therefore, the crumpled porous films can serve as a new class of stretchable electronic materials with adjustable surface structures and material properties for varying target applications, such as robotics, prosthetics, virtual reality, and personalized healthcare (Figure 1c,d).

### 2.2 Structural and Composition Characterization of Crumpled Porous Graphene/NiS2 Nanocomposite

**Figure 2:**
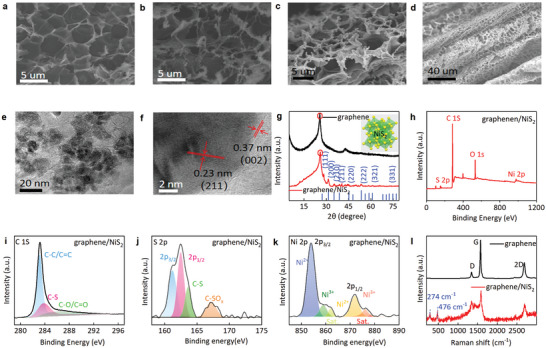
Characterizations of the LIG foam and crumpled porous graphene/NiS_2_ nanocomposite. Scanning electron microscopy images of a) LIG foam, porous graphene/NiS_2_ nanocomposite b) before and c) after the transfer, followed by d) the release of the pre‐strain to yield crumped porous graphene/NiS_2_ nanocomposite. e) Transmission electron microscopy (TEM) and f) high‐resolution TEM (HRTEM) of the crumpled porous graphene/NiS_2_ nanocomposite. g) X‐ray diffraction (XRD) patterns of LIG and crumpled porous graphene/NiS_2_ nanocomposite, the inset showing the schematic crystal structure of NiS_2_. h) Full survey X‐ray photoelectron spectroscopy (XPS) spectra of crumpled porous graphene/NiS_2_ nanocomposite. High‐resolution i) C *1s*, j) S *2p*, k) Ni *2p* XPS spectra of the crumpled porous graphene/NiS_2_ nanocomposite. l) Raman spectra of LIG foam and crumpled porous graphene/NiS_2_ nanocomposite.

Characterization of the crumpled graphene/NiS_2_ nanocomposite with scanning electron microscope (SEM), transmission electron microscopy (TEM), X‐ray diffraction (XRD), X‐ray photoelectron spectroscopy (XPS), and Raman spectroscopy reveals the structure and material composition. With few‐layered graphene sheets interconnected to form a porous honeycomb cellular structure in the pristine LIG (**Figure** **2**a), NiS_2_ nanoparticles are uniformly grown on the surface of graphene sheets in the nanocomposite for enhanced accessible surface area and ion diffusion rate (Figure 2b). The typical surface morphology of the as‐prepared samples after transfer printing (Figure 2c) and the full release of the pre‐strain (Figure 2d) clearly reveals ripple‐like crumped structures on the surface of the nanocomposite film with well‐maintained 3D porous network structure to afford high stretchability and specific surface area.^[^ ^18^ ^]^ Thin graphene flakes with ripple‐like wrinkled structures observed in the TEM image (Figure 2e) are likely attributed to the thermal expansion during the pulsed CO_2_ laser irradiation. NiS_2_ nanoparticles with a relatively uniform size of ≈20 nm also show porous structures (Figure S5, Supporting Information) for increased specific surface area and shorter transport pathways for both electrons and electrolyte ions.^[^ ^19^ ^]^ The lattice spacing of ≈0.23 and 0.37 nm in the high‐resolution TEM (HRTEM) image corresponds to the distance between two neighboring (211) planes in NiS_2_ nanocrystals and (002) planes in graphitic materials, respectively (Figure 2f).

The broad diffraction peak located at *2θ* = 25.9 in the XRD pattern of the LIG foam (Figure 2g) corresponds to the graphite (002) plane,^[^ ^7^ ^]^ suggesting the high degree of graphitization in LIG. In addition to the typical peak originating from LIG, the porous graphene/NiS_2_ nanocomposite also shows other well‐defined diffraction peaks in the XRD pattern, which can be well indexed to the cubic phase of NiS_2_ crystals (JCPDS Card No. 11–0099, space group Pa‐3(205); *a* = *b* = *c* = 5.67Å; *α* = *β* = *γ* = 90°).^[^ ^20^ ^]^ The full survey XPS spectra of the porous graphene/NiS_2_ nanocomposite (Figure 2h) mainly show carbon, sulfur, and nickel, along with a certain amount of oxygen resulting from the oxygen reaction on the surface of the nanocomposite. The C *1s* XPS spectrum shows three major peaks with binding energies at 283.2, 283.9, and 286.9 eV (Figure 2i), corresponding to C─C, C─S, and C─O peaks,^[^ ^21^ ^]^ respectively. Moreover, two dominant peaks at 853.9 and 871.8 eV in the S *2p* spectrum (Figure 2j) correspond to S *2p_3/2_* and S *2p_1/2_*, while two weak peaks at 161 and 163.9 eV correspond to a very small amount of NiS and organic sulfur, respectively.^[^ ^22^ ^]^ The high‐resolution Ni *2p* in the nanocomposite fits with Ni *2p_1/2_* (871.8 eV) and Ni *2p_3/2_* (853.9 eV) spin‐orbit peaks and two shakeup satellite peaks at 878.9 and 862.5 eV (labeled as “Sat.”).^[^ ^22^ ^]^ Furthermore, three characteristic peaks at ≈1351 (D bond), 2580 (G bond), and 2700 cm^−1^ (2D bond) in the Raman spectrum of the LIG and porous graphene/NiS_2_ nanocomposite (Figure 2l) further confirm the formation of crystalline graphene. The D/G intensity ratio of ≈0.15 and 0.31 indicates a high degree of graphene structures in the as‐prepared samples. Additionally, two prominent peaks at 274 and 476 cm^−1^ correspond to NiS_2_ in porous graphene/NiS_2_ nanocomposite, indicating the successful growth of NiS_2_ on the LIG scaffold.^[^ ^23^ ^]^

### 2.3 Physicochemical Properties of the Crumpled Porous Graphene/NiS_2_ Nanocomposite

**Figure 3:**
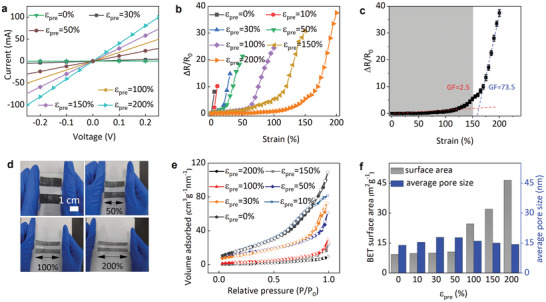
Physicochemical properties of porous graphene/NiS_2_ nanocomposite with different levels of the pre‐strain *ε_pre_* ranging from 0 to 200%. a) The linear relation current–voltage (*I*–*V*) curves suggesting the ohmic contacts between porous graphene foam and NiS_2_ nanoparticles. b) The electromechanical properties of the nanocomposite with various *ε_pre_*. c) The normalized resistance change (*ΔR/R_0_*) of the porous graphene/NiS_2_ nanocomposite with *ε_pre_* of 200% as a function of the tensile strain from 0 to 200%. Error bars represent standard deviations over ten measurements. d) Photograph of the crumpled porous graphene/NiS_2_ nanocomposite with *ε_pre_* of 200% under an applied strain ranging from 0 to 200%. e) Nitrogen adsorption/desorption isotherms of porous graphene/NiS_2_ nanocomposite with different *ε_pre_* and the corresponding f) Brunauer–Emmett–Teller (BET) surface area and the average pore size.

As the level of crumpled structure depends on the pre‐strain *ε_pre_*, the crumpled porous graphene/NiS_2_ nanocomposite is prepared with *ε_pre_* ranging from 0 to 200%. The linear current–voltage (*I*–*V*) curves of the crumpled porous graphene/NiS_2_ nanocomposite (**Figure** **3**a) indicate good ohmic contacts formed between NiS_2_ nanoparticles and the LIG foam scaffold. Moreover, the increased slope of the *I*–*V* curve with the increasing *ε_pre_* highlights the enhanced conductive pathways due to the increased effective contact area. Compared with the sheet resistance of 38 Ω sq^−1^ for the pristine LIG foam (*ε_pre_* of 200%), porous graphene/NiS_2_ nanocomposite and its crumpled form exhibit reduced values to 33 and 28 Ω sq^−1^, suggesting enhanced electrical conductivity from the direct growth of metallic NiS_2_ on the 3D networks scaffold. The sheet resistance value of the crumpled porous graphene/NiS_2_ nanocomposite with a pre‐strain *ε_pre_* of 200% is smaller than previously reported LIG, LIG foams with a variety of metals (Au nanocrystals),^[^ ^13^ ^]^ and metal oxides (Co_3_O_4_, MnO_2_).^[^ ^24^ ^]^ Furthermore, the normalized resistance change (*ΔR/R_0_*) of the crumpled porous graphene/NiS_2_ nanocomposite from increased *ε_pre_* exhibits enhanced stretchability and reduced changes with the increasing applied strain (Figure 3b). Meanwhile, the calibration curve of crumpled graphene/NiS_2_ nanocomposite films with *ε_pre_* of 200% is piecewise linear in two regions with extremely high gauge factor (*GF*, *GF = (ΔR/R_0_)/ε*) of 2.5 for strain 0–140%, and 73.5 for strain 140–200% (Figure 3c,d). Although large GF is often preferred for practical application, overlarge GF under an applied strain ranging from 140% to 200% can lead to a mass of small cracks in the crumpled graphene/NiS_2_ nanocomposite films, which would lead to negatively affect the electronic conductivity and electromechanical performance. Therefore, the excellent working range of the crumpled graphene/NiS_2_ nanocomposite films with *ε_pre_* of 200% is 140%, and the nanocomposite films can fully recover to its original state under small strains.

The pre‐strain *ε_pre_* can also be used to control the Brunauer–Emmett–Teller (BET) surface area and the pore size distribution. First of all, the hysteresis loop in the nitrogen adsorption/desorption isotherms of the porous graphene/NiS_2_ nanocomposite with different pre‐strain levels *ε_pre_* ranging from 0 to 200% indicates porous structures in all samples (Figure 3e). As the pre‐strain *ε_pre_* increases from 0 to 200%, the surface area calculated from the Barrett–Joyner–Halenda (BJH) method gradually increases from 158 to 217 m^2^ g^−1^, with the average pore size increased first and then decreased. The increased surface area with *ε_pre_* can be attributed to the increased wavy structure formed in the crumped porous graphene/NiS_2_ nanocomposite. Furthermore, the water contact angle decreases from 40° for the pristine LIG foam to 31° for the crumped porous graphene/NiS_2_ nanocomposite with *ε_pre_* of 200% (Figure S6, Supporting Information), suggesting improved surface wettability due to the electrodeposited NiS_2_ nanoparticles for faster adsorption of ionic liquids and enhanced electrochemical energy storage.

### 2.4 Output Performance of the Crumpled Porous Graphene/NiS_2_‐Based Stretchable TENG

**Figure 4:**
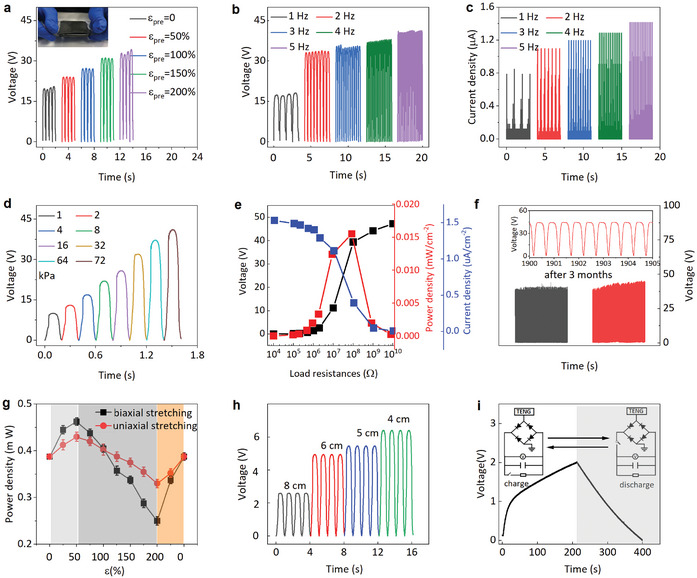
The electrical output performance of the intrinsically stretchable TENG is based on a top‐crumped porous graphene/NiS_2_ top electrode and a bottom‐stretchable PDMS electrode. a) The output voltage of the stretchable TENG (ɛ_pre_: 0, 50, 100, 150, and 200%), with the photograph of the stretchable TENG (ɛ_pre_ of 200%, dimension of 5 cm × 5 cm) shown in the inset. b) Output voltage and c) current of the stretchable TENG (ɛ_pre_ of 200%) as the frequency increases from 1 to 5 Hz. d) Output voltage of the stretchable TENG with increasing driving pressure. e) Changes in the output voltage, current density, and power density with the increasing external load resistance. f) Long‐term stability of the stretchable TENG over 2000 cycles of contact‐separation motions before and after 3 months. The inset shows a magnified view of the voltage plot after 3 months. g) The normalized output power density of the stretchable TENG as the applied biaxial and uniaxial tensile strain increases from 0 to 200% and followed by a full release. Error bars represent standard deviations over ten measurements. h) The output voltage of the stretchable TENG under different bending angles. i) Charging and discharging curve of a commercial capacitor charged by the TENG driven by a linear mechanical motor through a commutator rectifier (discharge at 5 ηA), with the equivalent circuit diagram shown in the inset.

The crumped porous graphene/NiS_2_ nanocomposite with highly conductive and stretchable properties as a top electrode can be combined with a bottom triboelectric polydimethylsiloxane (PDMS) layer (with four PDMS cylindrical spacers) to yield an intrinsically stretchable TENG (**Figure** **4**a). As the TENG utilizes contact electrification coupled with electrostatic induction to convert mechanical energy into electricity (Figure S7, Supporting Information), triboelectric charges generated from cyclic mechanical motion flow through external circuits to maintain electrostatic equilibrium, generating an alternating current output. The efficiency of the TENG depends on the difference in the electron affinities of the two electrode materials and the microstructures of the contact surfaces, so the level of the pre‐strain *ε_pre_* plays a critical role in the performance of the stretchable TENG. As the pre‐strain *ɛ_pre_* increases from 0 to 200%, the output voltage of stretchable TENG (at a frequency of 2 Hz) monotonically increases from 19 to 34 V, which is attributed to increased surface roughness and effective contact area (Figure 4a). Therefore, the pre‐strain *ε_pre_* of 200% is chosen in the following studies unless specified otherwise. As the deformation frequency increases from 1 to 5 Hz (at a pressure of 72 kPa), the output current (and voltage) increases from 0.8 to 1.4 µA (and from 18 to 42 V) (Figure 4b,c), resulting from higher flow rate of charges. In addition, the output voltage also increases from 10 to 41 V as the pressure increases from 1 to 72 kPa (Figure 4d), which is attributed to the increased contact area between the two triboelectric electrodes. By using a load resistor from 10 to 10 GΩ, the output voltage (or current density) of the TENG sharply increases (or decreases) to result in a peak output power density of 1.6 × 10^−2^ mW cm^−2^ at a resistance of 2 mΩ (Figure 4e). The crumped porous graphene/NiS_2_‐based stretchable TENG also demonstrates excellent mechanical stability with negligibly small changes in the output voltage over 3 months (Figure 4f).

As the biaxial (or uniaxial) strain increases from 0 to 50% and then to 200%, the maximum output power of the device first increases from 3.9 × 10^−1^ to 4.6 × 10^−1^ (or 4.3 × 10^−1^) mW but then reduces to a minimum value of 2.5 × 10^−1^ (or 3.3 × 10^−1^) mW with 64% (or 85%) output power retention (Figure 4g). The initial increase results from increased effective contact area between the crumpled graphene/NiS_2_ and PDMS layer upon stretching, while the subsequent decrease is likely attributed to the increased resistance of the crumpled graphene/NiS_2_ electrode. The highly elastic property of the TENG also allows the recovery of the structure and output power density to the initial state after the tensile strain is fully released. The stretchable TENG also shows a stable maximum output power density over 1000 cycles under various biaxial and uniaxial strains (Figure S8, Supporting Information). As the bending at a radius of 8/6/5/4 cm, the output voltage (or peak output power density) increases from 2.7 to 6.1 V (or from 0.6×10^−4^ to 3.4 × 10^−4^ mW cm^−2^) (Figure 4h), resulting from the higher input mechanical energy and the effective contact area. By using a bridge rectifier, the intrinsically stretchable TENG (at 72 kPa) can charge a commercial capacitor (NCC200, 2 V, 100 ηF) (Figure 4i) and low‐power consumer electronics. The excellent output performance of the crumpled porous graphene/NiS_2_‐based stretchable TENG compares favorably with the previously reported TENGs^[^ ^25^, ^26^, ^27^ ^]^ (Table S1, Supporting Information).

### 2.5 Electrochemical Performance of the Crumpled Porous Graphene/NiS_2_‐Based MSCAs

**Figure 5:**
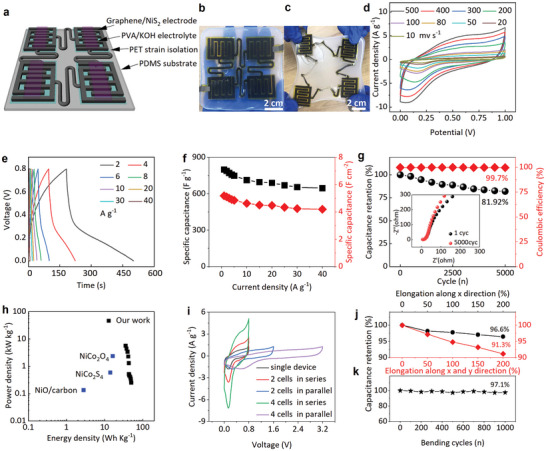
Electrochemical characteristics of the stretchable all‐in‐one microsupercapacitor arrays (MSCAs) based on crumped porous graphene/NiS_2_ at room‐temperature of 28 °C. a) Schematic showing the stretchable MSCA with the island‐bridge layout. Photograph of stretchable MSCAs b) before and c) after 50% biaxial stretching. d) Cyclic voltammograms (CV) curves at various scan rates and e) galvanostatic charge–discharge (GCD) plots at various current densities of the MSC cell. f) Rate performance of the MSC cell with sequentially varied current density. g) Cycling stability and Coulombic efficiency of the MSC cell, with the corresponding electrochemical impedance spectroscopy (EIS) after 1 and 5000 cycles shown in the inset. h) Ragone plot of the gravimetric energy density versus gravimetric power density for the MSC from this work in comparison with other MSCs previously reported in the literature. (i) CV curves of MSCs connected in serial and/or parallel compared with a single MSC cell (at 10 mV s^−1^). Capacitance retention of the stretchable all‐in‐one MSCA with four MSCs connected in series upon j) uniaxial/biaxial stretching from 0% to 200% and k) bending at 180° over 1000 cycles.

With superior structural and electronic properties, the crumpled porous graphene/NiS_2_ nanocomposite can act as interdigitated electrodes, current collectors, and serpentine interconnections in an all‐in‐one planar micro‐supercapacitor array (MSCA) with polymeric gel electrolyte (PVA/KOH) and island‐bridge layout (**Figure** **5**a). Compared with pristine LIG‐based MSC cells with nearly rectangular shapes as double‐layer capacitors (Figure S9a, Supporting Information), crumpled porous graphene/NiS_2_‐based MSC shows a distinct pair of redox peaks during the anodic and cathodic sweeps in cyclic voltammetry (CV) (Figure 5d). The redox peaks result from the faradic oxidation of NiS_2_ with the alkaline electrolyte: NiS_2_ + 3OH^−^ ↔ NiS_2_(OH)_3_ + 3e^−^.^[^ ^21^ ^]^ As the scan rate increases, the current density increases and the reduction/oxidation peaks move to more positive/negative potential (Figure 5d), indicating the relatively low internal resistance and good high‐rate capability of the electrode. In addition, the galvanostatic charge–discharge (GCD) curves of the porous graphene/NiS_2_‐based MSC at different current densities show significantly increased discharging time compared to that of pristine LIG‐based MSC cells (Figure S9b, Supporting Information), further suggesting larger charge capacity from NiS_2_ nanoparticles (Figure 5e). Furthermore, nonlinear charge/discharge curves further corroborate rapid Faradaic pseudocapacitance reactions to govern charge storage. Despite the strong pseudocapacitive behavior suggested by the GCD curves, the charge/discharge curves are approximately symmetric, indicating a superior reversible redox reaction and good Coulombic efficiency. The specific areal (or gravimetric) capacitance *C_a_* (or *C_g_*) of the porous graphene/NiS_2_‐based MSC only decreases from 5.19 F cm^−2^ (or 799 F g^−1^) to 4.20 F cm^−2^ (or 646 F g^−1^) (with rate capability of 80.1%) as the current density increases from 1 to 40 A g^−1^ (Figure 5f). Collectively, the porous LIG/NiS_2_‐based MSC with high specific areal (or gravimetric) capacitance and maximum energy density compares favorably over the other LIG‐based MSCs (Figure S9c and Table S2, Supporting Information). The GCD charging and discharging curves of the crumpled porous graphene/NiS_2_‐based MSC in the temperature range from −20 to 60 °C are approximately symmetric, indicating a good electrochemical capacitive characteristic and excellent reversible redox reaction of the porous graphene/NiS_2_ electrodes (Figure S10a, Supporting Information). Meanwhile, the slight curvature in the discharge curve suggests a balanced contribution from both pseudocapacitance and the double‐layer capacitance. As the temperature increases from −20 to 60 °C, the charge storage capacity of the device is enhanced by ≈160% (Figure S10b, Supporting Information), which may be attributed to the fast reaction kinetics of the crumpled porous graphene/NiS_2_ electrodes and improved mobility of the electrolyte ions. These results demonstrate that the prepared MSCAs based on the crumpled porous graphene/NiS_2_ can be used in a wide temperature range. The composite‐based MSC can also retain ≈81.92% of its initial capacitance even after 5000 CV cycles, with superior Coulombic efficiency of 99.7% (at a constant current density of 3 A g^−1^) to indicate excellent cycling stability (Figure 5g). The comparison in the electrochemical impedance spectroscopy (EIS) of the device before and after 5000 charge–discharge cycles only shows a slightly increased faradaic charge‐transfer resistance (*R_ct_*) and the equivalent series resistance (*R_s_*). The good stability of the device over cycling results from the high electrical conductivity and excellent ion diffusion within the porous crumped porous graphene/NiS_2_ electrode. Considering the total device mass (i.e., electrodes, current collectors, and electrolyte), the nanocomposite‐based MSC delivers a maximum energy density of 49.89 Wh kg^−1^ at a power density of 0.25 kW kg^−1^, with the highest power density of 5.70 kW kg^−1^ at the energy density of 35.61 Wh kg^−1^. The power and energy densities of the device are superior to the previously reported MSCs in the literature, including NiCo_2_S_4_ (31.5 Wh kg^−1^ at 0.156 kW kg^−1^),^[^ ^28^ ^]^ NiCo_2_O_4_ (34.9 Wh kg^−1^ at 0.875 kW kg^−1^),^[^ ^29^ ^]^ and NiO/carbon (10.2 Wh kg^−1^ at 0.025 kW kg^−1^)^[^ ^30^ ^]^ as revealed in the Ragone plot (Figure 5h).

Connecting MSC cells in series and/or in parallel in the all‐in‐one planar MSCA can provide tunable output voltage and current. Compared with a single MSC cell with an output voltage window of 0.8 V, the MSCAs with two or four MSC cells connected in series can deliver an output working voltage window of 1.6 or 3.2 V with similar charge/discharge time (Figure 5i; Figure S11a, Supporting Information). Similarly, the output current of the MSCAs with two or four MSC cells connected in parallel is increased by the factor of two or four, with the charge/discharge time increased by two or four times as well (Figure 5i; Figure S11b, Supporting Information). Moreover, the overall capacitance of the MSCA is linearly proportional to the number of MSC cells in the array (Figure S11c,d, Supporting Information), demonstrating excellent structural uniformity and scalability.

The islands‐bridge layout together with the strain isolation design provided by a relatively rigid polyethylene terephthalate (PET) film between the MSC cell and stretchable PDMS substrate^[^ ^17^ ^]^ allows the MSCA to maintain stable electrochemical performance upon stretching (Figure 5b). The strain isolation and island‐bridge design allow the MSCA to be stretched 50% biaxially (Figure 5c). The combination of the intrinsically stretchable crumpled graphene/NiS_2_ nanocomposites with serpentine interconnects further increases the stretchability (Figure S12, Supporting Information). The stretchable MSCA with four MSC cells connected in parallel shows a small capacitance change of 3.4% (or 8.7%) even for uniaxial (or biaxial) stretching of 200% (Figure 5j) and it also retains more than 97.1% of its initial capacitance after bending of 180° over 1000 cycles. These results clearly demonstrate that the as‐prepared stretchable MSCAs exhibit high mechanical and electrochemical stability, which is highly promising for flexible electronics.

### 2.6 Sensing Performance of the Crumpled Porous Graphene/NiS_2_‐Based Wearable Sensors

**Figure 6:**
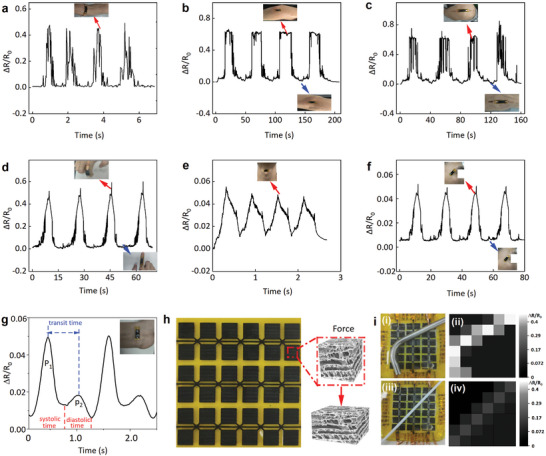
Characterization of wearable piezoresistive strain sensor based on crumpled porous graphene/NiS_2_ for monitoring human body activities. The relative resistance changes from the bending‐release movement of the a) ankle, b) elbow, c) knee, and d) fingers. The relative resistance changes from e) swallowing, f) eye blinking, and g) arterial pulse. h) Photograph of the as‐prepared 6 × 6 sensor array to detect pressure distribution from i) a heavy metal bar (top) and a lightweight plastic pipe (bottom) with the corresponding output shown in the right column.

As the crumpled porous graphene/NiS_2_ nanocomposite in the 3D network is sensitive to the deformation caused by external pressure/strain, its seamless and conformal contact with human skin with the help of a biocompatible liquid bandage (Nexcare, 3 m) allows for accurate and long‐term measurements of body motion. The strain sensor based on a single sensing unit attached to the joint (**Figure** **6**a–d) or muscle (Figure 6e–g) monitors the subtle motion from human activities. The porous 3D networked microstructure upon strain such as bending or stretching results in varied resistance of the crumpled porous graphene/NiS_2_ nanocomposite. Attaching the strain sensor (3.0 mm × 20.0 mm^2^) to the ankle noninvasively detects the human joint motion (Figure 6a) to monitor the walking frequency. Similarly, the sensor attached to the elbow (Figure 6b), knee (Figure 6c), and fingers (Figure 6d) can detect the bending‐release movement of the elbow, knee, and fingers. Besides the large motions, the wearable sensor can also detect subtle human activities, including swallowing (Figure 6e), eye blinking (Figure 6f), and arterial pulse (Figure 6g). The measured radial artery pulse exhibits a distinguishable systolic peak (“P_1_”) and diastolic peak (labeled as“P_2_”), with an average value of 72 beats per minute that is consistent with the measurement from a commercial sphygmomanometer (Omron, J760). In addition to excellent mechanical durability and stability over 55 000 loading‐unloading cycles (1 kPa at 4 Hz) (Figure S13, Supporting Information), the sensor fabricated from the low‐cost and scalable method can be facilely configured into an array of 6 × 6 to detect pressure distribution from a bent metal bar and a straight beverage straw. By employing the piezoresistive mechanism (Figure 6h), the applied external pressure increases the conductive pathways and reduces the total resistance. As a result, the as‐prepared 6 × 6 sensor array can detect the shape of the metal bar (and beverage straw) with the color contrast mapping to illustrate the distribution of pressure (Figure 6i).

**Figure 7:**
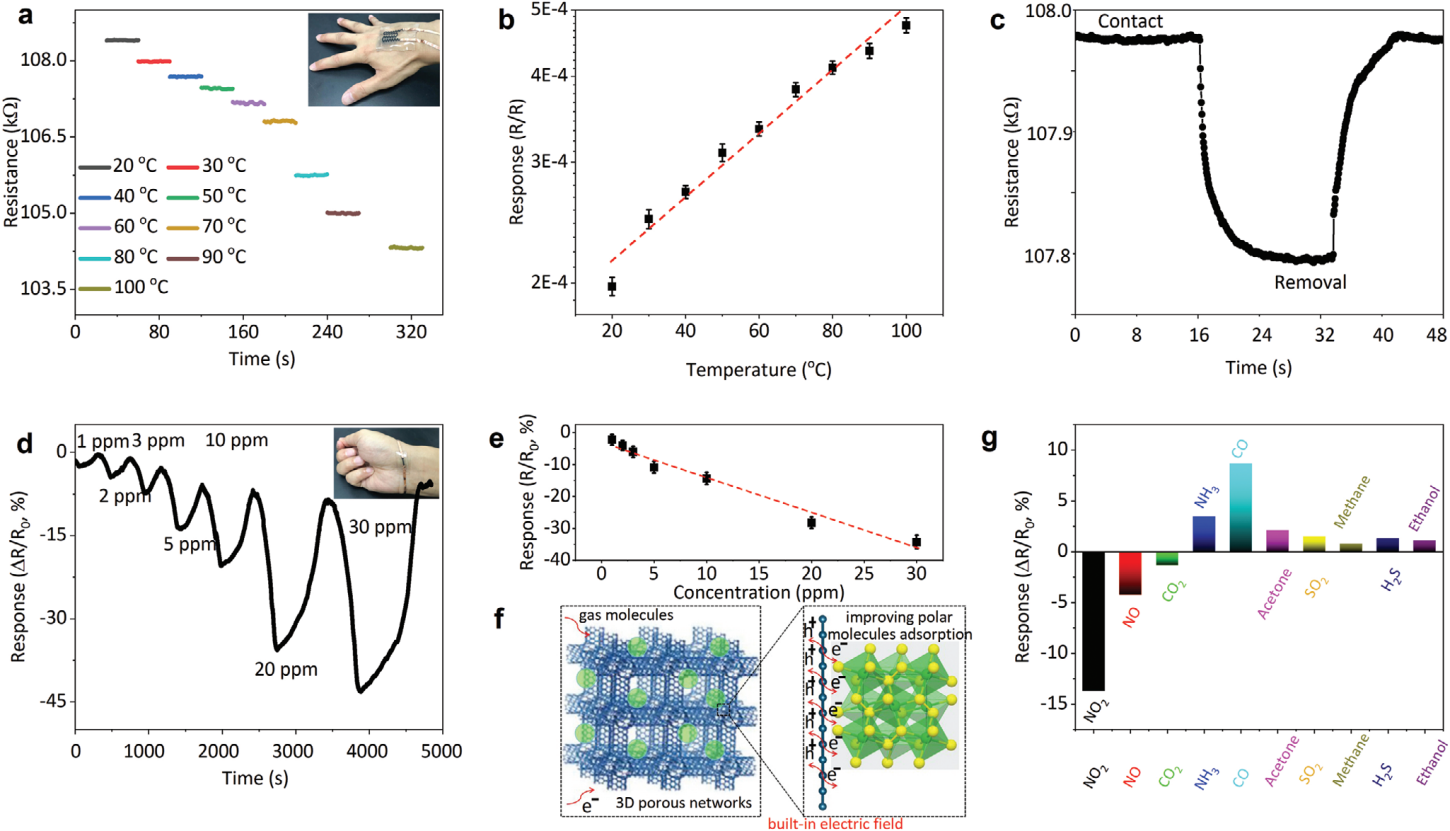
Characterization of wearable temperature and gas sensors based on crumpled porous graphene/NiS_2_. a) The real‐time resistance changes of the temperature sensor with stepwise increased temperatures from 20 to 100 °C, with the photograph of the sensor on the human skin shown in the inset. b) The corresponding calibration curve of the temperature sensor. Error bars represent standard deviations over ten measurements. c) Dynamic response of wearable temperature sensor upon contact and removal from the human body (room‐temperature of 28 °C). d) Real‐time sensing response of the gas sensor to NO_2_ from 1 to 30 ppm, with the photograph of a stretchable gas sensor on the wrist shown in the inset. e) The corresponding calibration curve with a linear relationship between the response and the NO_2_ concentration. Error bars represent standard deviations over ten measurements. f) Schematic showing the adsorption/desorption of gas molecules on the crumpled porous graphene/NiS_2_ surface. g) The selectivity of the stretchable NO_2_ gas sensor.

As the electron‐phonon scattering and thermal velocity of electrons in the nanocomposite increase with the rising temperature,^[^ ^31^ ^]^ the increased charge conductivity of the crumpled porous graphene/NiS_2_ in the serpentine shape can detect the corresponding temperature rise. With the temperature sensor calibrated by a commercial thermometer (Deli, LE505) in the range from 20 to 100 °C (**Figure** **7**a), the approximately linear relationship between the relative resistance change and the temperature leads to a sensitivity value of *S* = 3.38 × 10^−6^ °C^−1^ (R^2^ = 0.999). Attaching/removing the wearable temperature sensor on/from the human wrist measures a response/recovery time of 10.5/8.5 s (Figure 7c), which compares favorably with that of 15.5/12.5 and 10.5/9.5 s from other wearable temperature sensors based on LIG and indium oxide.^[^ ^31^, ^32^ ^]^

The crumpled porous graphene/NiS_2_ also allows it to detect NO_2_ with varying concentrations from 1 to 30 ppm at room‐temperature (Figure 7d). Meanwhile, the gas sensor exhibits a fast response/recovery time of 160/187 s to 1 ppm NO_2_ even at room‐temperature. The sensitivity of 1.12 ppm‐1 (R^2^ = 0.969) obtained from the linear fit between the response and NO_2_ concentration (Figure 7e) is higher than that of 0.65 ppm^−1^ (R^2^ = 0.953) from the LIG‐based gas sensor (Figure S14, Supporting Information). The crumpled porous LIG/NiS_2_‐based gas sensors with high sensitivity and rapid response/recovery also compare favorably over many previously reported NO_2_ gas sensors (Table S3, Supporting Information).^[^ ^33^, ^34^ ^]^ The high sensitivity of the gas sensor may be attributed to the unique 3D porous network structures of the nanocomposite and the high charge‐transfer kinetics from metallic NiS_2_ nanoparticles on the highly conductive LIG scaffold (Figure 7f). Moreover, a built‐in electric field is established at the interface of LIG foam and NiS_2_ nanoparticles due to their different work functions (4.1 and 3.5 eV for LIG and NiS_2_ measured by a Kelvin probe force microscopy), resulting in significantly improved adsorption of the polar gas molecules.^[^ ^14^, ^35^ ^]^ Furthermore, the response of the wearable gas sensor to 10 ppm of NO_2_ is much larger than that to 10 ppm of NO, CO_2_, NH_3_, CO, acetone, SO_2_, methane, H_2_S, and ethanol (Figure 7g), demonstrating excellent selectivity to NO_2_. Meanwhile, various decoupling strategies can be applied to multimodal sensors for improved accuracy in practical applications,^[^ ^36^, ^37^ ^]^ which will be leveraged in future studies for the demonstration of decoupling sensing based on the crumpled porous graphene/NiS_2_ nanocomposites.

### 2.7 Integration and Demonstration of the Crumpled Porous Graphene/NiS_2_‐Based Standalone Biophysical Sensing Platform

**Figure 8:**
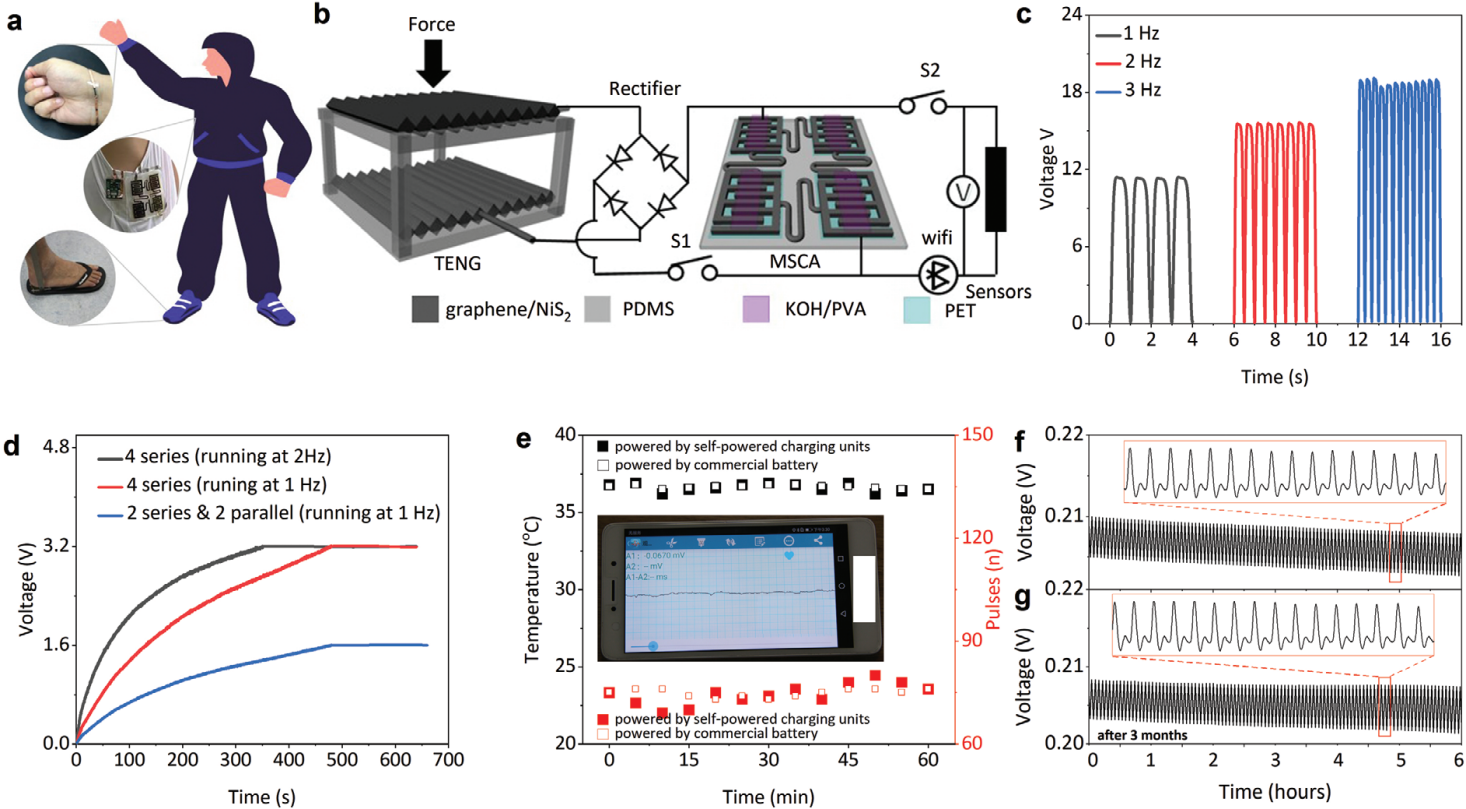
Integration and demonstration of a standalone stretchable device platform for continuous monitoring of the body temperature and pulse. a) Schematic and b) circuit diagram of the integrated standalone platform on the human subject. c) The output voltage from the crumpled porous graphene/NiS_2_‐based TENG shoe sole driven by a human subject (body weight of 80 kg) at varying running frequencies. d) The real‐time potential of the stretchable MSCAs with different numbers of MSCs connected in series/parallel charged by the TENG driven by a human subject under different running frequencies. e) Comparison in the measured body temperature and pulse at the wrist between the standalone stretchable device platform and the system powered by a commercial battery. Continuously monitored pulse wave signals over 6 h f) before and g) after 3 months from the standalone stretchable device platform under the same indoor environmental conditions.

The full integration of the crumpled porous graphene/NiS_2_‐based wearable biophysical sensors, TENG, and MSCAs with power management circuits and wireless transmission modules (Bluetooth chips, CC2541) provides a standalone biophysical sensing platform (**Figure** **8**a,b). The two TENGs attached to the shoes of a human subject (with a body weight of 80 kg) convert mechanical energy from human motion such as running at a frequency of 1, 2, or 3 Hz into electrical energy with a stable maximum output voltage of 11.4, 15.7, or 19.2 V (Figure 8c), corresponding to a peak output power density of 1.2 × 10^−1^, 2.2 × 10^−1^, or 3.3 × 10^−1^ mW cm^−2^. The harvested intermittent and alternating electrical energy from the insole TENGs is stored as electrochemical energy in the MSCAs through a bridge rectifier. As the human subject runs at a constant frequency of 1 or 2 Hz, the real‐time charging voltage of the stretchable MSCAs with four MSC cells connected in series (4S, 4 cells in series) or parallel (2P^*^2S, 2 cells in series and then in parallel) first rapidly and then slowly increases to the stable operating voltage of 3.2 or 1.6 V after 352 or 481 s (Figure 8d). Meanwhile, the charging takes 478 s when the stretchable MSCAs consist of two MSC cells connected in series and another two connected in parallel (at 1 Hz). These results demonstrate that the self‐powered charging units can efficiently harvest and store energy with an adjustable power output during running exercise.

The sustained power source provided by the wearable self‐powered charging units can drive low‐power wearable biophysical sensors and wireless transmission modules for long‐term continuous measurements. In the proof‐of‐the‐concept demonstration, the skin temperature and pulse signals from a healthy subject running on the treadmill (at 1 Hz) measured by the fully integrated standalone biophysical sensing platform are consistent with those from the integrated systems powered by a commercial battery (Nanfu, 1300 mAg, 1.5 V) during the 60 min exercise (Figure 8e). In particular, the measured skin temperature (or arterial pulse) of the healthy human subject with an average of 36.6 °C (or 74.5 bpm) and a standard deviation of 0.72 (or 0.63) from the fully integrated system agrees reasonably well with that of 36.7 ± 0.83 °C (or 74.8 ± 0.91 bpm) from the system powered by the commercial battery. Furthermore, the comparison in the continuously monitored radial artery pulse signal over 6 h before (Figure 8f) and after 3 months (Figure 8g) indicates stable distinguishable systolic peak (“P1”) and diastolic peak (labeled as“P2”), confirming the long‐term stability of fully integrated standalone biophysical sensing platform.

## 3 Conclusion

In summary, we demonstrated a facile and low‐cost fabrication approach for preparing 3D crumpled graphene/NiS_2_ nanocomposite patterns through laser direct writing combined with electrodeposition and the pre‐strain strategy. With high mechanical stretchability and electrical conductivity, the resulting functional nanomaterial composites have been demonstrated in a standalone stretchable sensing device that harvests mechanical energy with TENG and stores the converted electrical energy in MSCAs for driving various biophysical sensors. The mechanically stretchable the crumpled graphene/NiS_2_ nanocomposite patterns with versatile functions prepared by a facile fabrication approach hold great potential in health monitoring, personalized medicine, and human‐machine interfaces.

## Conflict of Interest

The authors declare no conflict of interest

## Author Contributions

K.W., H.C., and C.Z. performed conceptualization, investigation, wrote, reviewed, and edited the final manuscript. Q.J., J.G., W.W., C.Z., and J.W. performed investigation, data curation, wrote, reviewed, and edited the final manuscript.

## Supporting information



Supporting Information

## Data Availability

The data that support the findings of this study are available from the corresponding author upon reasonable request.
