# Supplementary material for: Facile Design of Highly Stretchable and Conductive Crumpled Graphene/NiS2 Films for Multifunctional Applications
Source: Small Methods. 2025 Jan 9;9(4):2401965. doi: 10.1002/smtd.202401965 (PMC12020346; doi:10.1002/smtd.202401965)
Supplement: Supplementary file 1 — Supporting Information [file SMTD-9-2401965-s001.docx]

***Supporting Information***

**Facile Design of Highly Stretchable and Conductive Crumpled Graphene/NiS_2_ Films for Multifunctional Applications**

Kangwei Weng^a,b^, Qiji Jing^a,b^, Jindong Gao^a^, Weiguo Wang^b^, Chen Zhang^a^, Jun Wang^a*^, Huanyu Cheng^c*^, Cheng Zhang^a*^

^a^ *Fujian Provincial Key Laboratory of Functional Marine Sensing Materials, College of Material and Chemical Engineering, Minjiang University, Fuzhou 350108, P. R. China*

^b^ *School of Materials Science and Engineering, Fujian University of Technology, Fuzhou, Fujian 350506, P. R. China*

^c^ *Department of Engineering Science and Mechanics, Materials Research Institute, Pennsylvania State University, University Park, Pennsylvania 16802, USA*

^*^To whom correspondence should be addressed. E-mail: wjnaf@163.com (W.J), huanyu.cheng@psu.edu (H.C.), and zhangcheng@mju.edu.cn (Z.C.)

**Experimental section**

**Materials:** The chemicals used in this work were of analytical reagent grade. Polyimide (PI) film, NiCl_2_‧6H_2_O, CH_4_N_2_S, KOH, Nafion, PVA (molecular weight: 75 000-80000 g/mol), and polydimethylsiloxane (PDMS) were purchased from Aladdin Chemistry Co., Ltd. (Shanghai, China). Solutions were freshly prepared with deionized water.

**Fabrication of crumped porous** **graphene/NiS_2_ nanocomposites:** After laminating a cleaned PI film on a spin-coated thin elastomer substrate (PDMS), laser scribing of the PI with a computer-controlled CO_2_ laser (10.6 μm with pulse duration of ~ 14 μs, laser power of 3.8 W) under ambient conditions prepared the porous graphene foam. NiS_2_ nanoparticles were electrodeposited on the LIG scaffold (2.0 × 3.0 cm^2^) as the working electrode in a three-electrode electrochemical system, where a Pt sheet (1.0 × 1.0 × 0.1 cm^3^) and a saturated Ag/AgCl were used as the counter and reference electrodes, respectively. An aqueous solution with 25 mM NiCl_2_‧6H_2_O and 1 M CH_4_N_2_S was prepared as the electrolyte. NiS_2_ nanoparticles were electrodeposited by cyclic voltammetry (CV) with the potential range from -1.4 to 0.1 V at a scan rate of 3 mV s^-1^ for 1 000 s. Next, the prepared samples were washed thoroughly with deionized water and annealed at 220°C in an argon gas atmosphere for 1 h with a heating rate of 5°C min^-1^. Carefully laminating the LIG/NiS_2_ film supported by the Si chip substrate to a pre-stretched acrylic elastomer film (3M VHB 4905), followed by peeling off the Si chip and releasing the pre-strain, formed crumpled structure in the LIG/NiS_2_ film on VHB. PDMS film was spin-coated on the crumpled LIG/NiS_2_ film, and then the crumpled LIG/NiS_2_ film supported by PDMS film obtained after peeling off the VHB film by heating at 120 for 1h.

**Fabrication of crumped porous graphene/NiS_2_-based TENG:** Two PDMS films with crumped porous graphene/NiS_2_ nanocomposites as the top and bottom electrodes were separated by four PDMS cylinders as the spacer, resulting in crumped porous graphene/NiS_2_-based stretchable TENG (length of 5 cm and width of 5 cm).

**Fabrication of crumped porous graphene/NiS_2_-based MACAs:** First, the PVA/KOH gel electrolyte was prepared by adding 3.58 g of KOH into 60 mL of deionized water followed by adding 6 g of PVA power, which was heated to 95°C under stirring until the solution became clear. Next, the PVA/KOH gel electrolyte was drop-coated on top of the crumped porous graphene/NiS_2_ interdigitated electrodes on the PDMS substrate using a syringe. After 10 min to fully diffuse the electrolyte into the porous structure, the sample was further heated to and kept at 35°C for 12 h to remove excess water in the electrolyte. Laminating a cleaned PDMS film on top of the gel electrolyte as an encapsulation layer yielded the crumped porous graphene/NiS_2_ nanocomposites-based MSCAs.

**Fabrication of crumped porous graphene/NiS_2_-based biophysical sensor**: The crumped porous graphene/NiS_2_-based piezoresistive pressure/temperature sensors were obtained by connecting a serpentine/ rectangular crumped porous graphene/NiS_2_ electrode with two Cu lead wires on the PDMS substrates, followed by packaging with a thin PDMS layer. The crumped porous graphene/NiS_2_-based gas sensor was obtained by connecting needlelike crumped porous graphene/NiS_2_ gas-sensitive region with two Cu lead wires on the PDMS substrates.

**Characterization**: The morphology of samples was characterized by an optical camera (D750, Nikon, Japan), scanning electron microscopy (SEM, Hi-tachiS3400, Japan), and transmission electron microscopy (TEM, JEOL-2100F, Japan). The structural and electronic properties of samples were characterized by X-ray diffraction patterns (XRD, Thermo ARL X’TRA, Switzerland), X-ray photoelectron spectroscopy (XPS, Ulvac-Phi, Inc., Japan), and Raman spectra (HORIBA, LabRAM HR 800, France). Electrochemical performance measurements were performed on an electrochemical workstation (CHI 660D, Chenhua Instruments, China). The voltage, current, and resistance were measured using a multimeter (DMM 7510, Keithley, Cleveland, OH, USA).

**Gas-sensing testing:** Before the start of the gas-sensing experiment, dry air was passed into the test chamber for one hour to ensure stable resistance. Next, the test gas (including NO_2_, NO, CO_2_, NH_3_, CO, acetone, SO_2_, Methane, H_2_S, and Ethanol) was delivered by controlling a dynamic gas distribution instrument (Tanggao Electric Technology, GC400, China) with a constant flow of 200 sccm. The resistances of gas sensors were captured using a multimeter system (DMM 7510, Keithley, Cleveland, OH, USA).

**Electrochemical calculations**: The capacitance *C* of electrode materials and devices was calculated from the GCD curves using the following equation:

*C=(I×Δt)/ΔV,*

where I, Δt, and ΔV are the discharge current, time, and potential range, respectively. The gravimetric (or areal) specific capacitance C_g_ (or C_A_) was calculated as the ratio of the capacitance to the mass of the active material (or area of the electrode/device). The energy and power densities based on the mass of active materials were then evaluated from GCD curves using the following equations:

$E_{g}=\frac{1}{2}\times C_{g}\times\frac{(\Delta V)^{2}}{3600}$，$E_{A}=\frac{1}{2}\times C_{A}\times\frac{(\Delta V)^{2}}{3600}$

$P_{\text{g}}=\frac{E_{g}}{\Delta t}\times3600$, $P_{\text{A}}=\frac{E_{A}}{\Delta t}\times3600$

**Table S1**: Performance comparison between our and recently reported stretchable TENGs.

| Electrode materials | peak output power density | Elongation (%) | Ref. |
| --- | --- | --- | --- |
| crumpled porous graphene/NiS_2_ // PDMS | 1.6 × 10^-2^ mW cm^-2^ | 200 | This work |
| PAAm-LiCl hydrogel // PDMS or VHB | 0.35 × 10^-2^ mW cm^-2^ | 1160 | 1 |
| 3-ply-twisted stainless steel/polyester fiber blended yarn | 0.85 × 10^-2^ mW cm^-2^ | 60 | 2 |
| PDMS-silicone/ionic solution | 0.18 ×10^-2^ mW cm^-2^ | 60 | 3 |
| PDMS // crumped Au | 0.22 mW cm^-2^ | 100 | 4 |
| PDMS // crumped graphene | 0.25 mW cm^-2^ | 120 | 5 |

**Table S2**: Performance comparison between our and recently reported supercapacitors.

| Electrode materials | Specific areal (or gravimetric) | Rate capability (the  capacitance retention) | Maximum energy density | Ref |
| --- | --- | --- | --- | --- |
| crumpled porous graphene/NiS_2_ | 5.19 F cm^-2^ (799 F g^-1^) at 1 A g^-1^ | 80.1% (the current incersed from 1 40 40 A g^-1^) | 49.89 Wh kg^-1^ at a power density of 0.25 kW kg^-1^ | This work |
| Co_3_O_4_@LIG | 3.0 mF cm^-2^ (143 F g^-1^ at 1 A g^-1^) | 71.5% (the current incersed from 1 40 40 A g^-1^) | 19.9 Wh kg^-1^ at a power density of 0.5 kW kg^-1^ | 6 |
| TiO_2_-graphene | 6.8 mF cm^-2^ at 5 μA | 47.1% (the current inceresed from 5 μA to 20 μA) | 0.22 μWh/cm^2^ and 39 μW/cm^2^ | 7 |
| Laser-scribed graphene (LSG) | 3.05 mF cm^−2^  at 16.8 mA cm^−3^ | 60% ( the current inceresed from 16.8 mA cm^−3^ to 1.84ｘ10^4^ mA cm^−3^) | 2.1 mWh cm^-3^ at 0.01 V s^-1^; 1.37 mWh cm^-3^ at 1 V s^-1^ | 8 |
| graphene | 80.7 μF at 200 mV s^-1^ | 97.8% (the scan rate inceresed from 200 mV s^-1^ to 1000 mV s^-1^) | 0.14 mWh cm^-3^ at a power density of 495 W cm^-3^ | 9 |
| PANI/nitrogen-doped graphene quantum dots | 261.85 mF cm^-2^ at 0.5 mA cm^-2^ | 97.55% (the current inceresed from 0.5 mA cm^-2^ at 1 mA cm^-2^) | 11.40 μWh cm^-2^ at 640 μWcm^-2^ | 10 |

**Table S3**: Performance comparison between our and recently reported NO_2_ sensors.

| Functional materials | Sensitivity (ppm^-1^) | Recover time (s) | Recover time (s) | Ref |
| --- | --- | --- | --- | --- |
| crumpled porous graphene/NiS_2_ | 1.12 | 160 | 187 | This work |
| Sulfonated rGO | 0.443 | 675 | 850 | 11 |
| ethylenediamine-modified rGO | 0.159 | 600 | 800 | 11 |
| MoS_2_ | 0.32 | 170 | 1630 | 12 |
| MoS_2_@rGO | 0.06 | 360 | 720 | 13 |
| rGO/Co_3_O_4_ | 0.03 | 300 | 2400 | 14 |
| rGO | 0.05 | 240 | 240 | 15 |


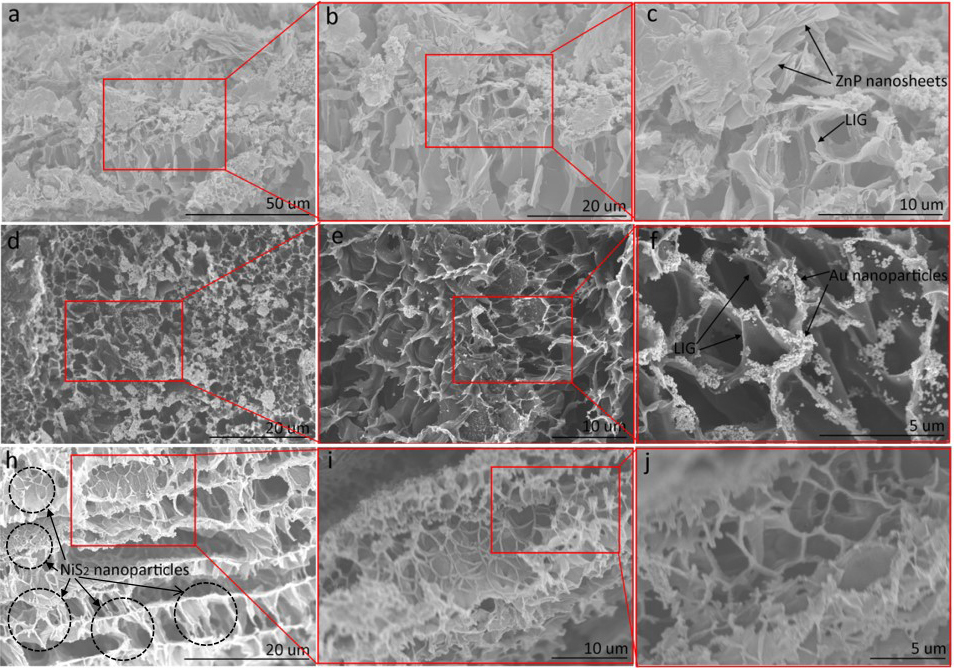


**Figure S1**: Scanning electron microscopy (SEM) images of LIG-based nanocomposite. (a-c) Side-view SEM images of crumpled porous graphene/ZnP nanocomposites prepared by surface spray coating and laser scribing (*Nano Energy*, 2021, 81, 105609). ZnP nanosheets are mainly anchored near the surface, as ZnP nanosheets with large size cannot be loaded deep into the LIG foam during the spray coating process. (d-f) Top-view SEM images of crumpled porous graphene/Au nanocomposites prepared by laser scribing (*Appl. Phys. Rev.* 2022, 9, 011413). Au nanoparticles are mainly formed at the top of LIG foam since the inside precursor materials cannot be effectively irradiated and converted due to the decreased power density of the laser with the increasing depth of the LIG substrate. (h-j) Side-view SEM images of crumpled porous graphene/NiS_2_ nanocomposites prepared by laser writing and electrodeposition. NiS_2_ nanoparticles uniformly grow on both the surface and inside the LIG scaffold due to the diffusion of ions in the electrolyte into the porous LIG scaffold during the electrodeposition process.


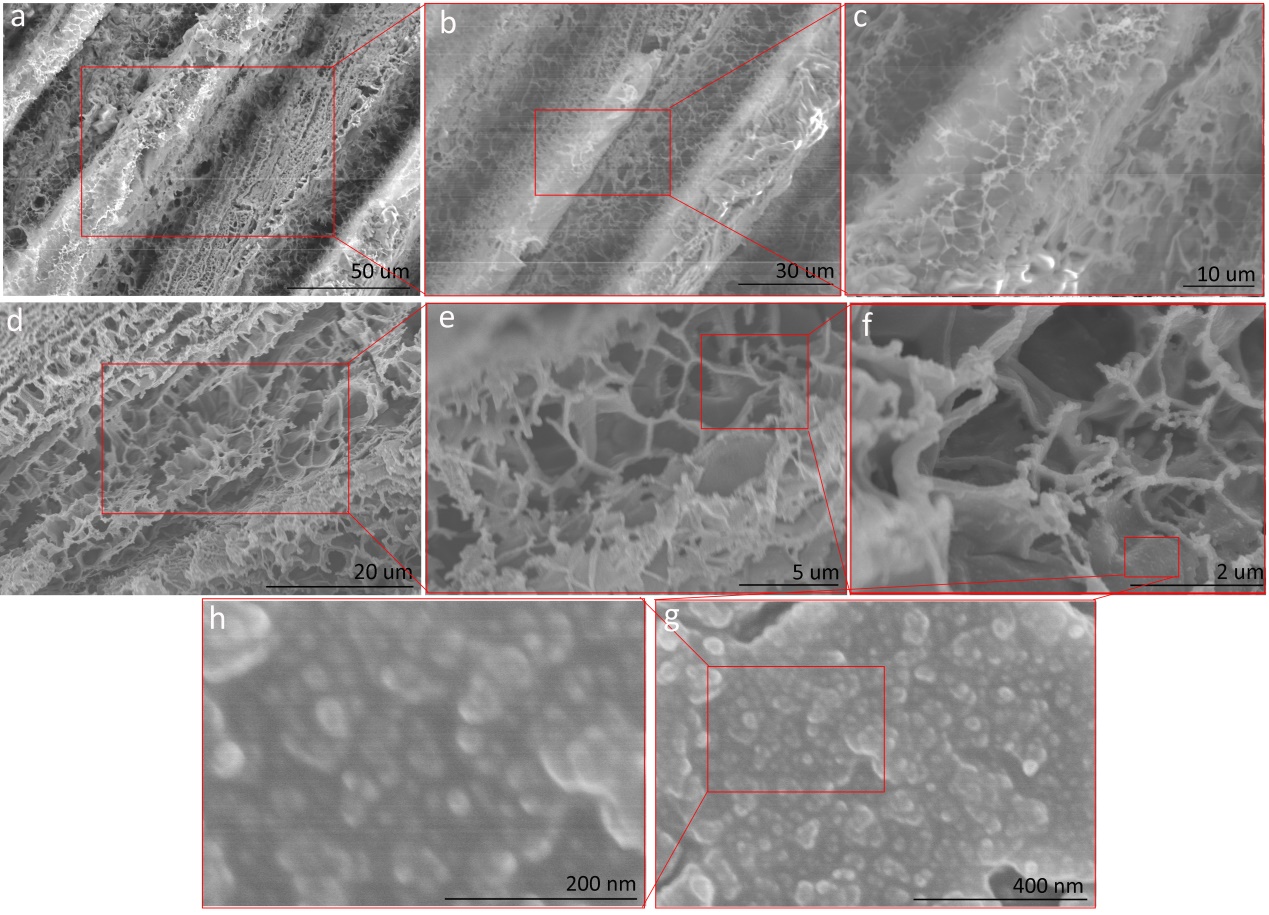


**Figure S2**: SEM images of crumpled porous graphene/NiS_2_ nanocomposites under (a-c) 0% and (d-h) 100% elongation to show the robust interface (without delamination).

**
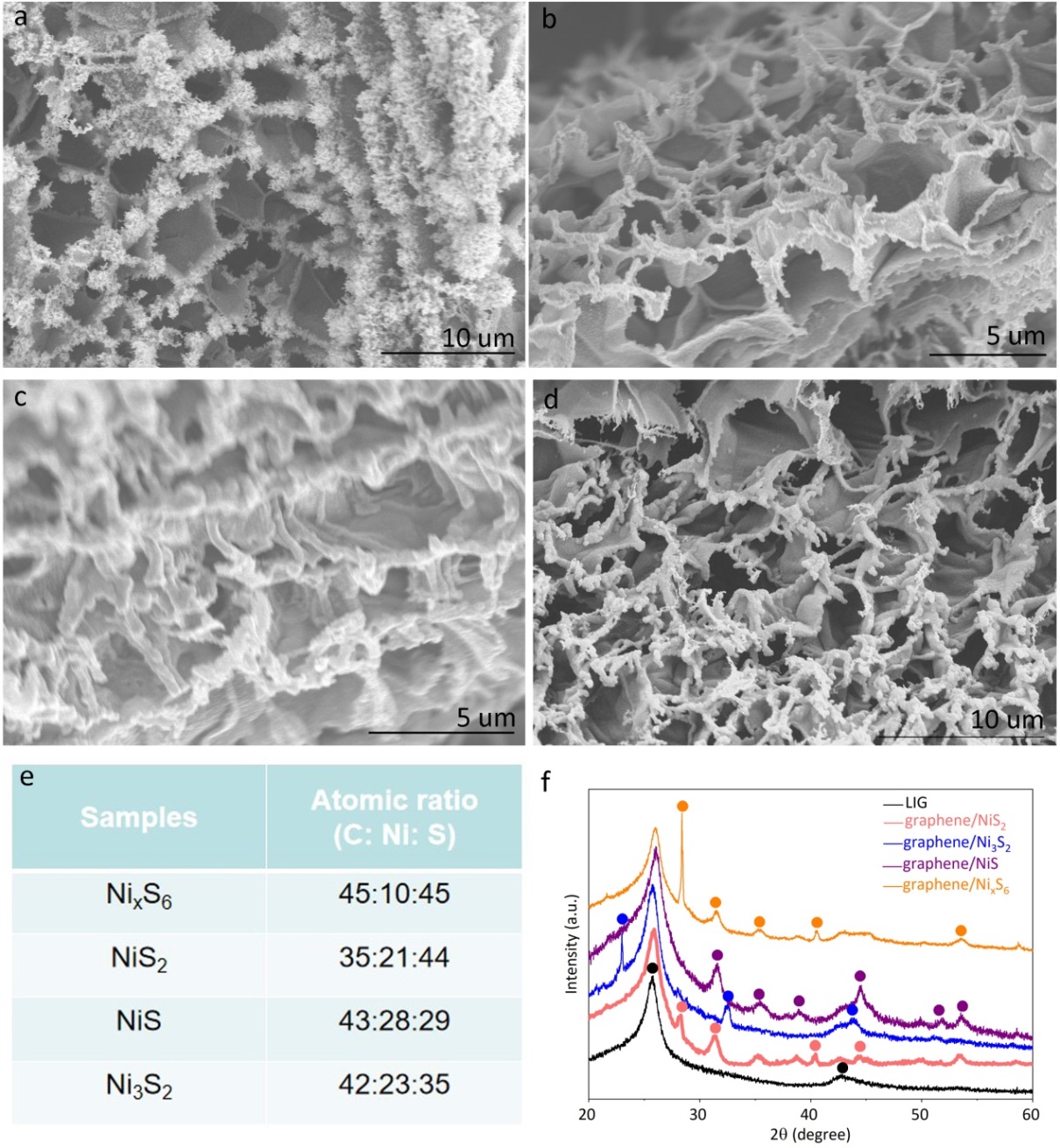
**

**Figure S3:** SEM images of (a) LIG/Ni_x_S_6_, (b) LIG/NiS_2_, (c) LIG/NiS and (d) LIG/Ni_3_S_2_ nanocomposites prepared by adjusting the atomic percentage of Ni and S in the solution. The atomic percentage of Ni and S in the solution are 1:60/1:40/1:20/1:10 for direct growth of Ni_x_S_6_/NiS_2_/NiS/Ni_3_S_2_ on the LIG foam through electrodeposition. (e) The atomic ratio and (f) XRD patterns of LIG/Ni_x_S_6_, LIG/NiS_2_, LIG/NiS, and LIG/Ni_3_S_2_ nanocomposites. The JCPDS card of the Ni_x_S_6_, NiS_2_, NiS, and Ni_3_S_2_ in the prepared LIG-based nanocomposites are 51-0718, 11-0099, 65-0395, and 44-1418, respectively. The atomic percentage of S is much larger than that of Ni in the solution during the electrodeposition process, which can be attributed to the higher growth rate of Ni ions than the S ions during the electrodeposition process.


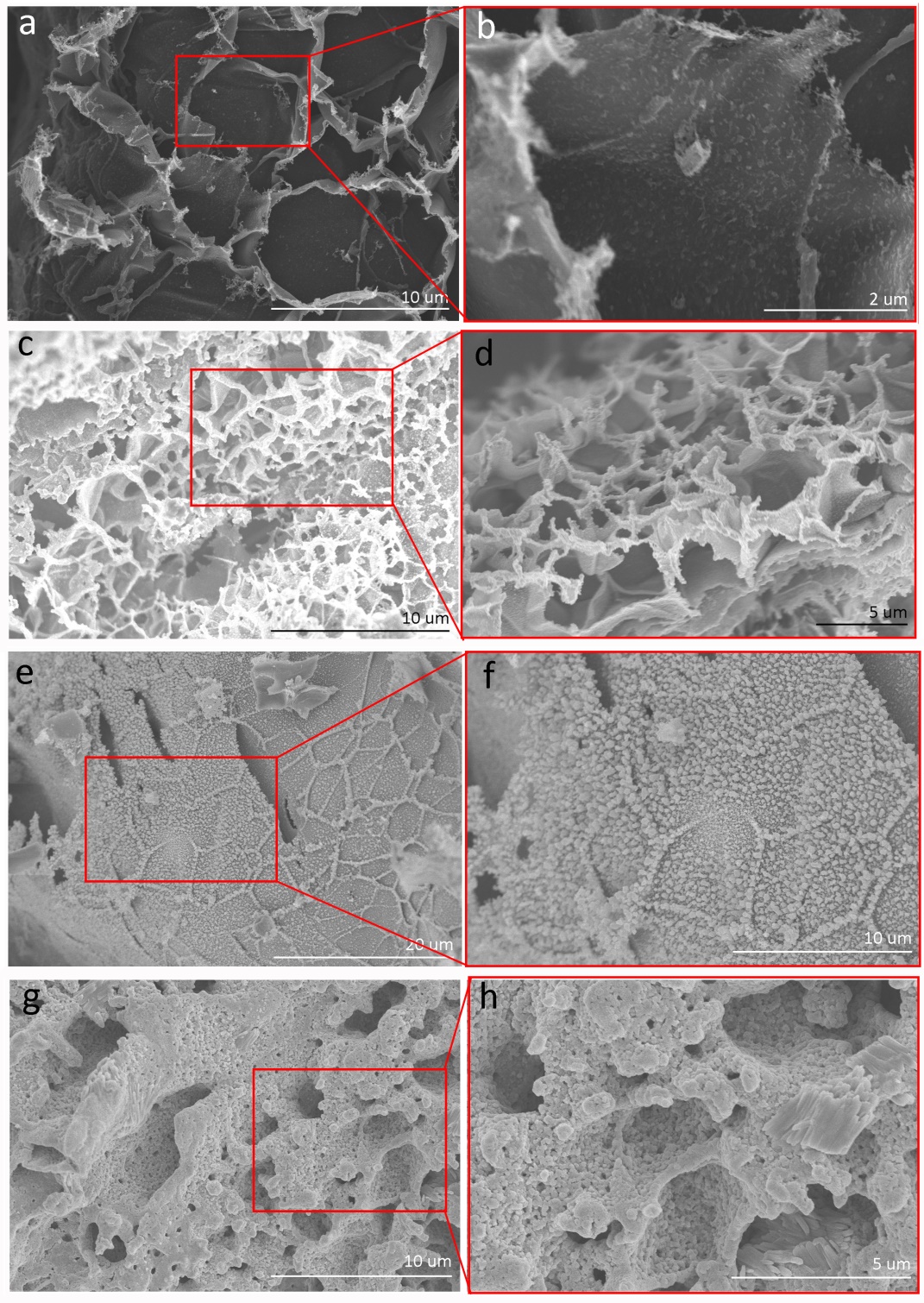


**Figure S4**: SEM images of LIG/NiS_2_ nanocomposite with different morphologies prepared by adjusting the electrodeposition time of (a-b) 500 s, (c-d) 1000 s, (e-f) 1500 s, and (g-h) 2000 s. The particle size and distribution density of the NiS_2_ increase with the increasing electrodeposition time.


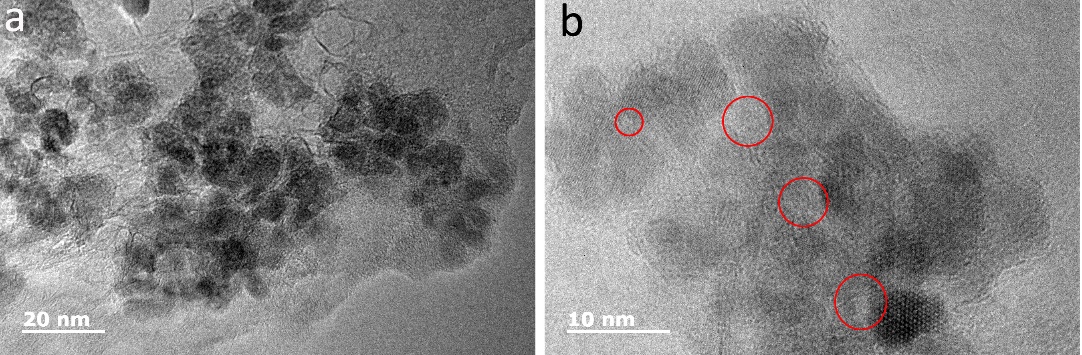


**Figure S5**: (a) Transmission electron microscopy (TEM) images and (b) high-resolution TEM (HRTEM) image of crumpled porous graphene /NiS_2_ nanocomposite samples. The highly porous feature of the NiS_2_ nanoparticles is clearly revealed by showing notable contrast difference between the hollow and solid parts in the HRTEM image.


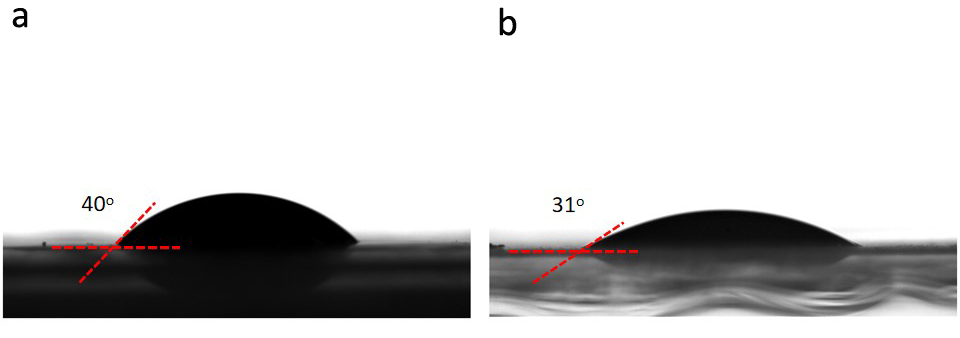


**Figure S6**: Water droplet on the surface of (a) pure LIG and (b) crumpled porous graphene /NiS_2_ nanocomposite samples.


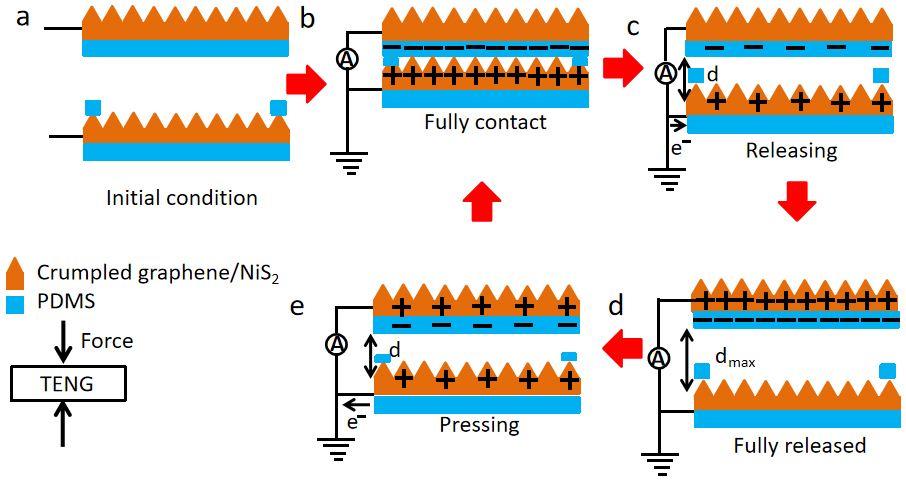


**Figure S7:** Schematic showing the working principle of the crumpled porous graphene/NiS_2_ nanocomposites-based TENG under cyclic compressive force. (a) When the bottom and top electrodes are separated by the spacer at the initial state, there is no electrical output. (b) Under a compressive force, electrostatic charges are generated and distributed on both the top and bottom electrodes. (c) The gradual release of the compressive force allows the electrons to flow from the top to the bottom electrode via electrostatic induction. (d) The device recovers to its original state when the compressive force is fully released, resulting in the maximum output voltage. (e) Once the device is pressed again, electrons flow back until the device returns to the full-contact state.


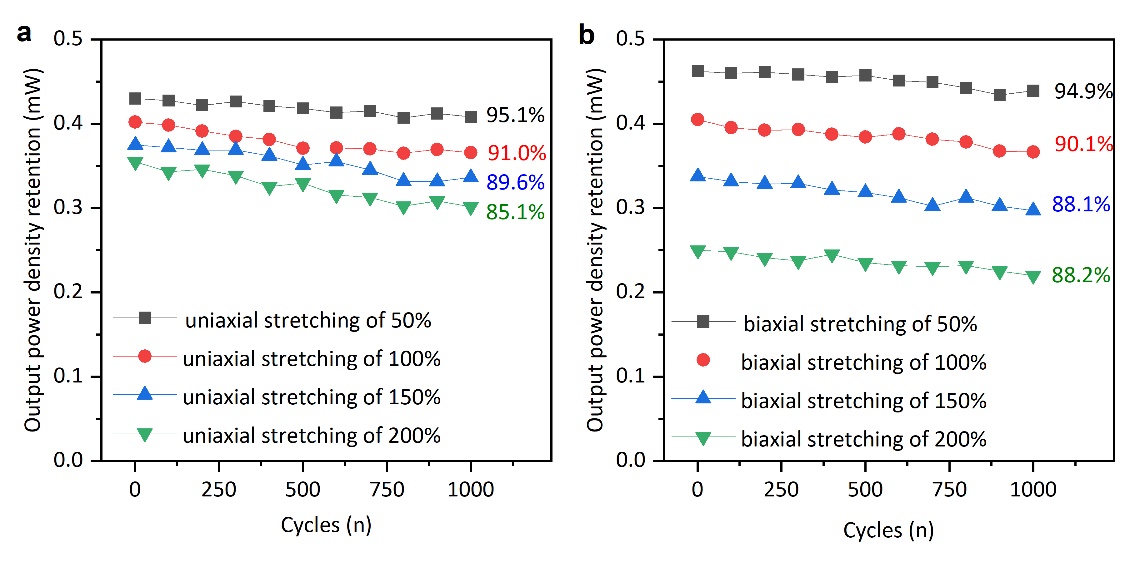


**Figure S8:** The maximum output power density retention of the stretchable TENGs under different (a) uniaxial and (b) biaxial strains over 1000 cycles to show the long term stability.


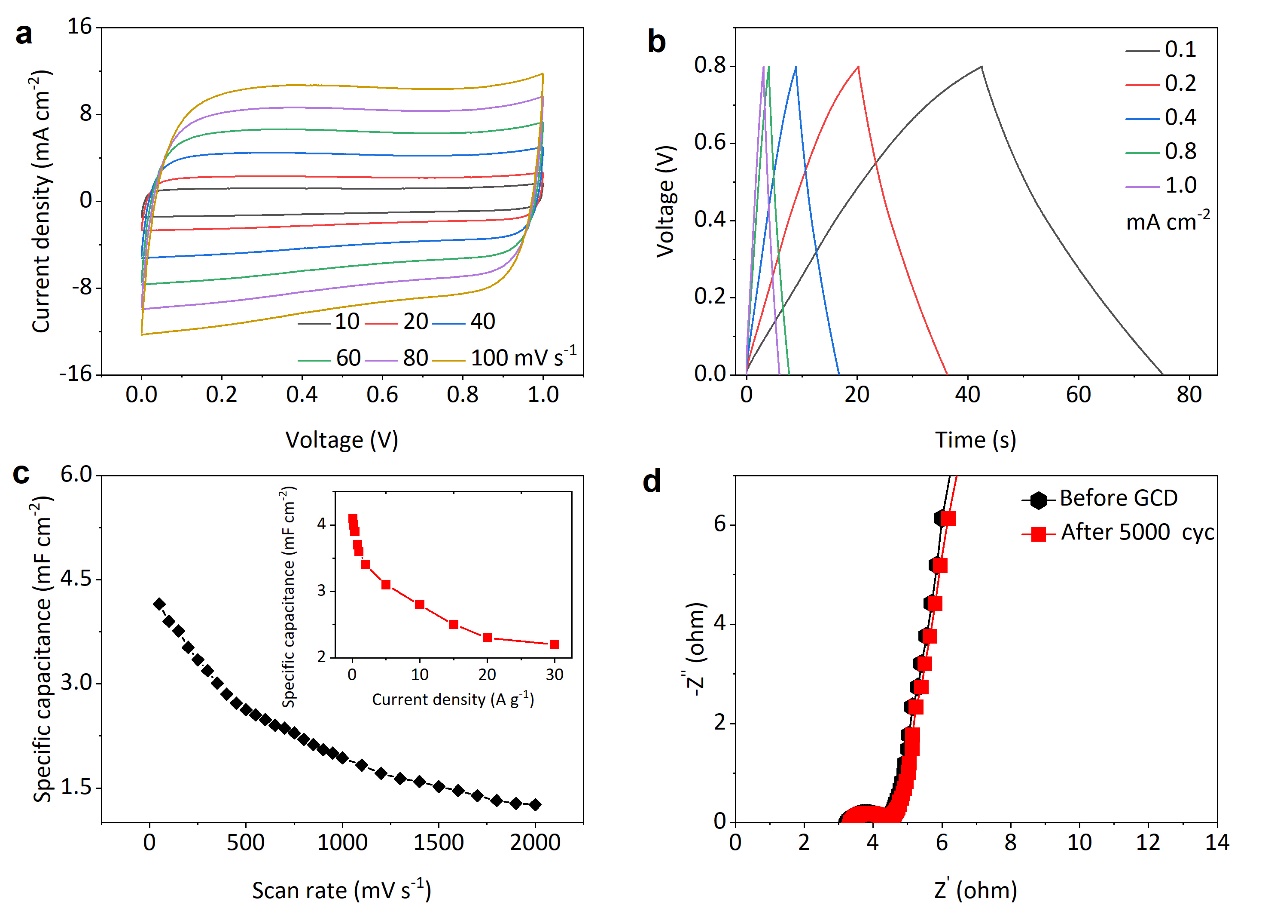


**Figure S9:** Electrochemical behavior of pristine LIG foam-based MSC. (a) CV curves of the MSC at various scan rates. (b) GCD curves of the MSC at various current densities. (c) Specific capacitance of the MSC as a function of the scan rate (or current density as shown in the inset). (d) The comparison in the electrochemical impedance spectra (EIS) before and after 5,000 charge-discharge cycles.


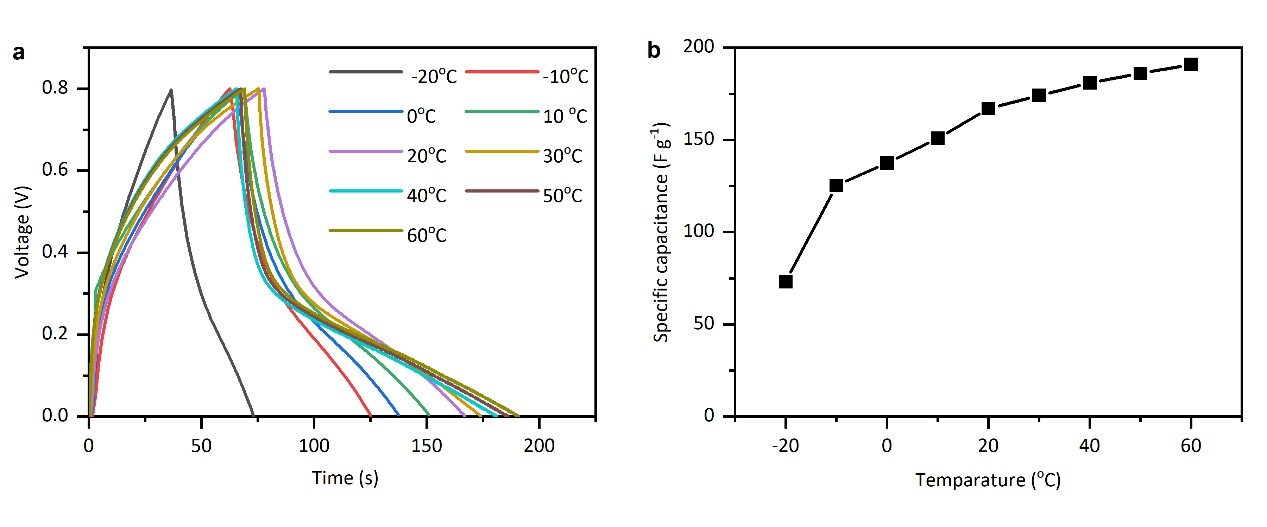


**Figure S10:** (a) GCD curves of the MSC cell at different temperatures (at a current density of 5 A g^-1^), and (b) the corresponding specific capacitance values.

**
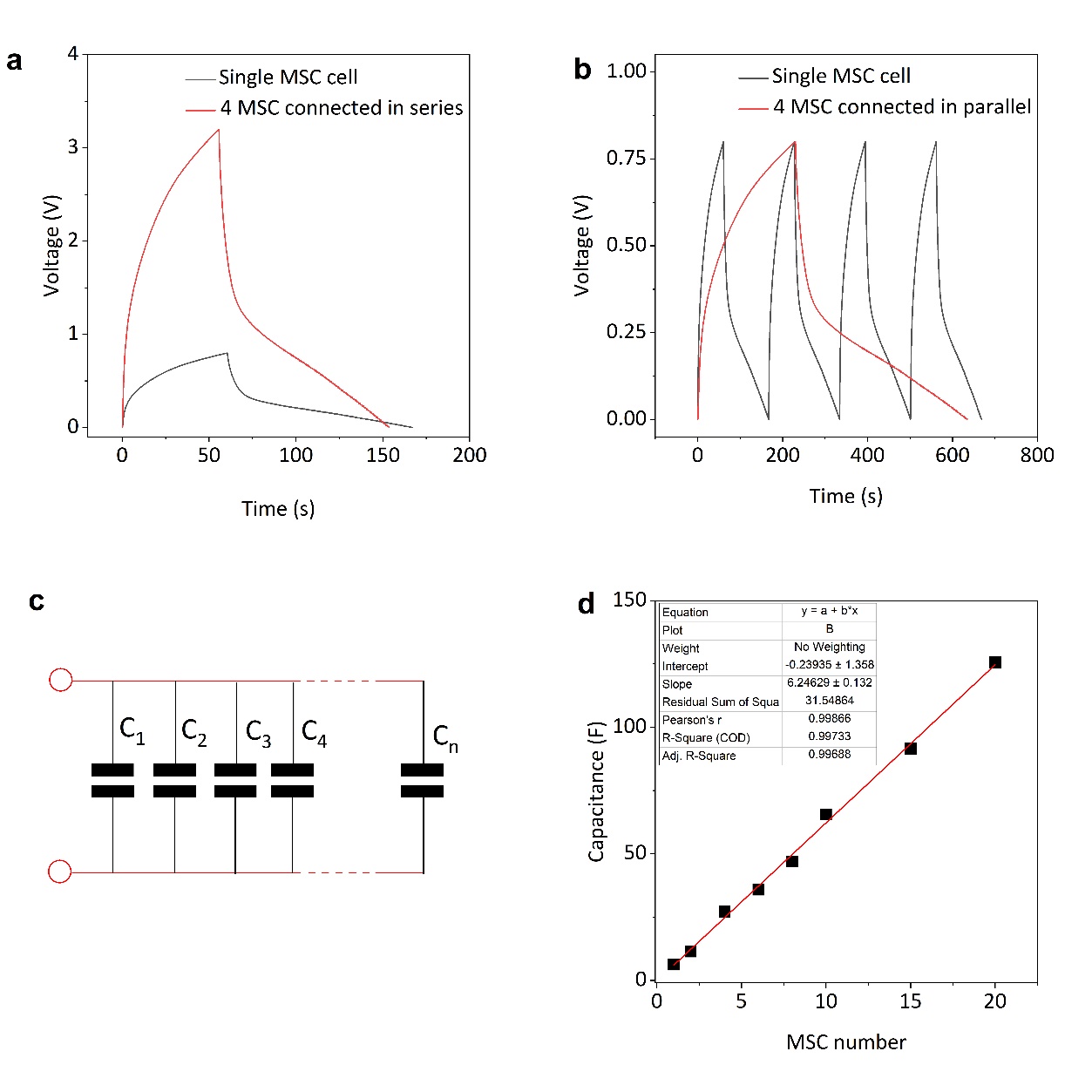
**

**Figure S11:** Electrochemical and mechanical properties of the all-in-one planar MSCAs with serial and parallel connections. Real-time collected CV curves of a single (black) and the all-in-one MSCA (red) with (a) serial and (b) parallel connections. (c) The equivalent circuit diagram of the four devices connected in parallel. (d) The relationship between the overall capacitance from the all-in-one MSCA and the number of devices connected in parallel.


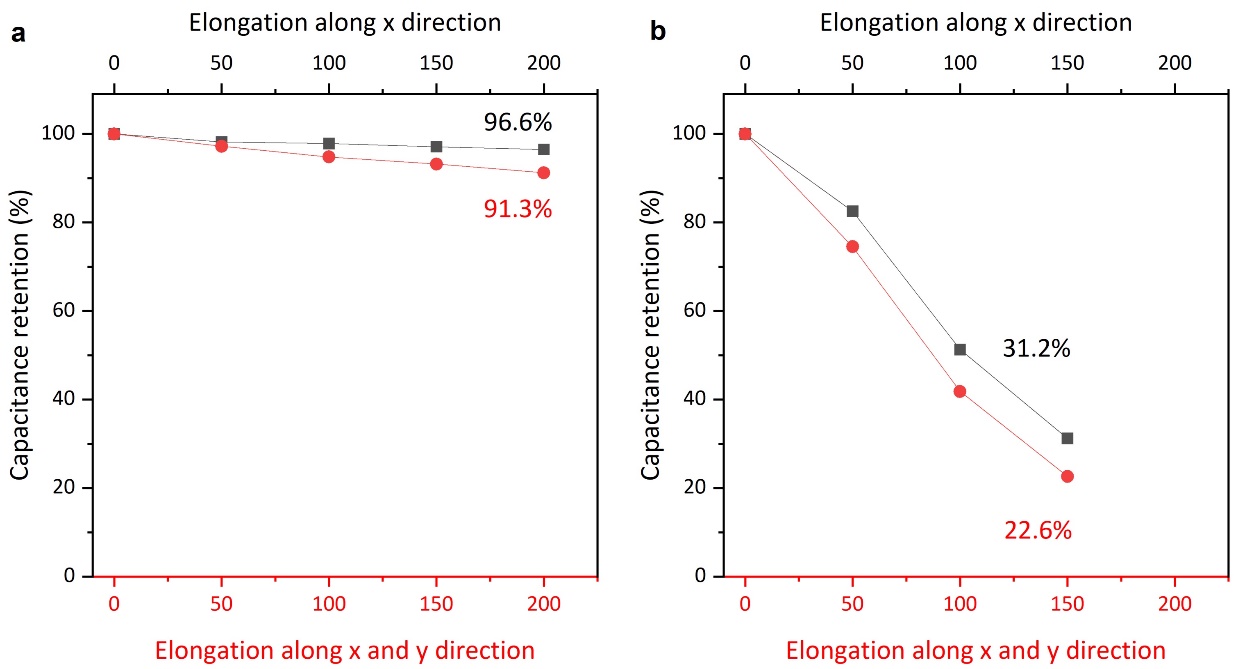


**Figure S12**: Capacitance retention of the stretchable all-in-one MSCA comprised of four MSCs interconnected in series upon uniaxial/biaxial stretching from 0% to 200% (a) with and (b) without serpentine interconnects.


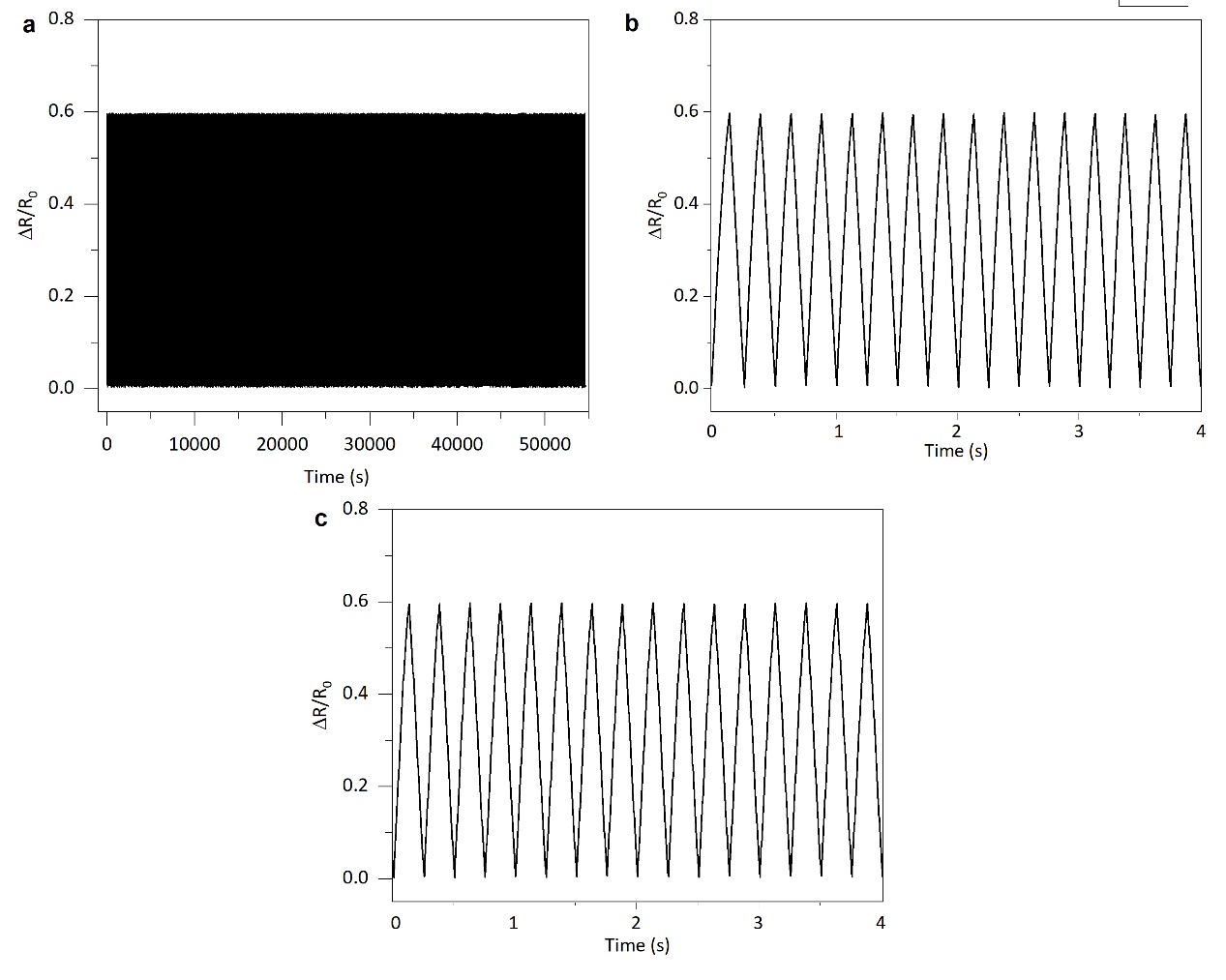


**Figure S13**: (a) The stability test of the crumpled porous graphene/NiS_2_-based piezoresistive pressure sensors over 55,000 cycles, with the dynamic response of the sensors (b) before and (c) after 55,000 cycles.


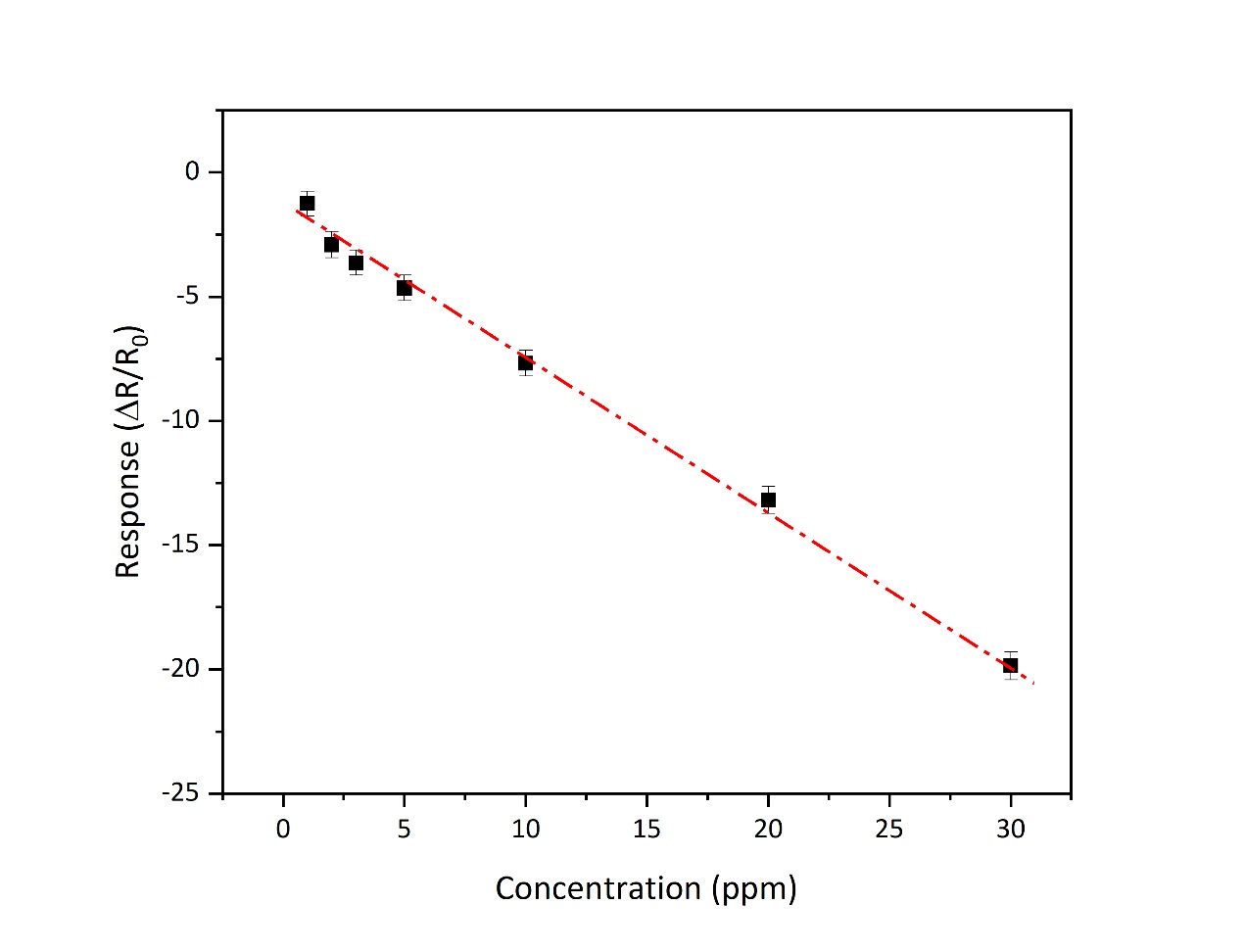


**Figure S14**: The corresponding calibration curve of a LIG-based gas sensor with a linear relationship between the response and the NO_2_ concentration room temperature of 28 °C. Error bars represent standard deviations over ten measurements.

**References**

[1] Xiong Pu, Mengmeng Liu, iangyu Chen, Jiangman Sun, Chunhua Du, Yang Zhang, Junyi Zhai, Weiguo Hu, Zhong Lin Wang, Ultrastretchable, transparent triboelectric nanogenerator as electronic skin for biomechanical energy harvesting and tactile sensing. *Sci. Adv*. **2017**, 3 (5), e1700015.

[2] Kai Dong, Yi-Cheng Wang, Jianan Deng, Yejing Dai, Steven L. Zhang, Haiyang Zou, Bohong Gu, Baozhong Sun, and Zhong Lin Wang, A Highly Stretchable and Washable All-Yarn-Based Self-Charging Knitting Power Textile Composed of Fiber Triboelectric Nanogenerators and Supercapacitors, *ACS Nano* **2017**, 11, 9, 9490–9499.

[3] Yang Zou, Puchuan Tan, Bojing Shi, Han Ouyang, Dongjie Jiang, Zhuo Liu, Hu Li, Min Yu, Chan Wang, Xuecheng Qu, Luming Zhao, Yubo Fan, Zhong Lin Wang, Zhou Li, A bionic stretchable nanogenerator for underwater sensing and energy harvesting, *Nat. Commun*. **2019**, 10 (1), 2695.

[4] Huamin Chen, Lin Bai, Tong Li, Chen Zhao, Jiushuang Zhang, Nan Zhang, Guofeng Song, Qiaoqiang Gan, Yun Xu, Wearable and robust triboelectric nanogenerator based on crumpled gold films, *Nano Energy*, **2018**, 46, 73-80.

[5] Huamin Chen, Yun Xua, Jiushuang Zhang, Weitong Wu, Guofeng Song, Enhanced stretchable graphene-based triboelectric nanogenerator via control of surface nanostructure. *Nano Energy*, **2019**, 58, 304-311.

[6] Xiaohong Ding, Ruilai Liu, Jingyun Zhao, Jiapeng Hu, Jinjin Wu, Cheng Zhang and Jing Lin, In situ formation of Co_3_O_4_ nanocrystals embedded in laser-induced graphene foam for high-energy flexible micro-supercapacitors, *Dalton Trans.*, **2022**, 51, 2846.

[7] Laura Fornasini, Silvio Scaravonati, Giacomo Magnani, Alberto Morenghi, Michele Sidoli, Danilo Bersani, Giovanni Bertoni, Lucrezia Aversa, Roberto Verucchi, Mauro Riccò, Pier Paolo Lottici, Daniele Pontiroli, In situ decoration of laser-scribed graphene with TiO_2_ nanoparticles for scalable high-performance micro-supercapacitors, *Carbon*, **2021**, 176, 296-306.

[8] Maher F. El-Kady, Richard B. Kaner, Scalable fabrication of high-power graphene micro-supercapacitors for flexible and on-chip energy storage, *Nat. Commun*. **2013**, 4, 1475.

[9] Zhong–Shuai Wu, Khaled Parvez, Xinliang Feng, Klaus Müllen, Graphene-based in-plane micro-supercapacitors with high power and energy densities, *Nat. Commun*. **2013**, 4, 2487.

[10] Navaneeth Punnakkal, S Naneena, Shyam Lal C P, Aarathi Pradeep, Satheesh Babu T G, Punathil Vasu Suneesh, Nitrogen-doped graphene quantum dot embedded polyaniline for the fabrication of high-performance flexible supercapacitor with enhanced cycling stability,  *J. Energy Storage*, **2024**, 100, 113527.

[11] Wenjing Yuan, Anran Liu, Liang Huang, Chun Li, Gaoquan Shi, High-Performance NO_2_ Sensors Based on Chemically Modified Graphene, *Adv. Mater.*, **2012**, 25, 766-771.

[12] Wei Zheng, Yongshan Xu, Lingli Zheng, Chen Yang, Nicola Pinna, Xianghong Liu, Jun Zhang, MoS_2_ Van der Waals p-n Junctions Enabling Highly Selective Room-Temperature NO_2_ Sensor, *Adv. Funct. Mater.*, **2020**, 30, 2000435.

[13] Ning Yi, Zheng Cheng, Han Li, Li Yang, Jia Zhu, Xiaoqi Zheng, Yong Chen, Zhendong Liu, Hongli Zhu, Huanyu Cheng, Stretchable, ultrasensitive, and low-temperature NO_2_ sensors based on MoS_2_@rGO nanocomposites, *Mater. Today Phys.*, **2020**, 15, 100265.

[14] Bo Zhang, Ming Cheng, Guannan Liu, Yuan Gao, Lianjing Zhao, Shan Li, Yipei Wang, Fangmeng Liu, Xishuang Liang, Tong Zhang, Geyu Lu, Room temperature NO_2_ gas sensor based on porous Co_3_O_4_ slices/reduced graphene oxide hybrid, *Sens. Actuators B Chem.*, **2018**, 263, 387-399.

[15] Vineet Dua, Sumedh P Surwade, Srikanth Ammu, Srikanth Rao Agnihotra, Sujit Jain, Kyle E Roberts, Sungjin Park, Rodney S Ruoff, Sanjeev K Manohar, All-organic vapor sensor using inkjet-printed reduced graphene oxide, *Angew Chem. Int. Edit*., **2010**, 49, 2154-2157.

.
